# Effects of Adachi Rehabilitation Programme on older adults under long-term care: A multi-centre controlled trial

**DOI:** 10.1371/journal.pone.0245646

**Published:** 2021-02-12

**Authors:** Yoshihiko Baba, Chika Ooyama, Yasushi Tazawa, Masahiro Kohzuki

**Affiliations:** 1 Faculty of Medicine, Kyorin University School of Medicine, Mitaka, Japan; 2 Tohoku Medical and Pharmaceutical University Hospital, Sendai, Japan; 3 Sendai Orthopaedic Hospital, Sendai, Japan; 4 Department of Internal Medicine and Rehabilitation Science, Tohoku University Graduate School of Medicine, Sendai, Japan; Kurume University School of Medicine, JAPAN

## Abstract

**Objectives:**

We developed the Adachi Rehabilitation Programme (ARP), a community rehabilitation program. Under the supervision of professional caregivers, older adults cleaned and planted flowers in the park and they walked and shopped in the community. We examined the effects of ARP on individuals receiving small-group multifunctional at-home care at community facilities.

**Methods:**

This was a multi-centre controlled trial at thirteen small multifunctional at-home care facilities in Adachi, Tokyo. The primary outcomes of the study were daily step counts and timed up & go (TUG). Secondary outcomes included gait speed, step length, Barthel Index for Activities of Daily Living, Functional Independence Measure, Mini-Mental State Examination (MMSE) and EuroQOL 5 Dimension.

**Results:**

Ninety-six individuals at thirteen small multifunctional at-home care facilities were recruited for participation in December 2017. They were allocated to intervention (38) and control (40) groups. The average daily step count of the control group decreased from 852 to 727, but it increased by approximately 650 steps, from 990 to 1635, for the intervention group. Average TUG decreased from 16.1 s to 14.0 s and MMSE score increased from 15.9 to 16.3 for the intervention group, but a significant interaction was not found. On non-intervention home days, the daily step counts of the intervention group increased significantly from 908 steps to 1485 steps, while those of the control group decreased from 865 steps to 722 steps.

**Conclusions:**

ARP may have effectively increased the physical activity of older adults under long-term care by increasing motivation and changing behaviour.

## Introduction

Older adults compared to other adults have a higher risk of losing their physical function once they suffer from diseases or disorders [1]. The World Health Organization recommends moderate-intensity exercises of at least 150 minutes per week. This is estimated to be 3,000 steps per day [2], but many patients after stroke walk as few as 1,200 steps per day [3,4]. Systematic reviews of the effects of physical therapy on community-dwelling frail older adults show that exercise two to three times per week may improve physical activity [5–7], but an epidemiological survey has revealed that 90% of elderly adults in nursing homes exercise once per week or less [8]. Therefore, increasing physical activity through behavioural change and other measures may be a more practical approach to better health for older adults under long-term care.

The need for long-term care for older adults has been rising in many countries. As a result, long-term care insurance (LCTI) has been launched in Germany, Japan, South Korea, Luxembourg and the Netherlands as of 2016 [9]. The Ministry of Labour, Health and Welfare of Japan, in revising LCTI in 2003, proposed community rehabilitation to enhance activity and participation for older adults with chronic diseases. To implement community rehabilitation, several new care services were introduced in 2006, including small multifunctional at-home care (SMAC). These facilities offered community-based service, which was legislated to offer daily care and functional training in a home-like setting and in communication with local residents [10].

At community-based long-term care facilities, rehabilitation through daily living and social interaction in the community is encouraged, including walking in the community. Walking, in fact, is one of the most popular and feasible moderate-intensity physical activities for older adults [2]. Although there is no consensus on the minimum clinically important difference for steps of older adults under LCTI, research has suggested 599 to 1131 steps per day for those with chronic obstructive pulmonary disease [11], and 800 steps per day for those with multiple sclerosis [12]. It is important to develop a programme for older adults to increase their physical activity, not only during the intervention but also in their spare time. Based on mall walking [13]⁠ and horticulture therapy [14,15], we developed the Adachi Rehabilitation Programme (ARP). In ARP, older adults clean and plant flowers in the park and walk and shop in the community under the supervision of professional caregivers. In this study, we offered ARP once per week at SMACs in Adachi, Tokyo, to demonstrate that ARP would improve physical activities of older adults under long-term care.

## Methods

### Research design

This was a multifacility controlled trial to demonstrate the superiority of ARP over standard care. The trial was registered at University Hospital Medical Information Network centre (UMIN000028317), ethically reviewed and approved by [Name Blinded for Review] University Hospital Ethical Committee (2017-2-213 (Application-8996)) on 2 December 2017 and reported according to the Transparent Reporting of Evaluations with Nonrandomized Designs statement [16]. The protocol is registered at http://protocols.io (dx.doi.org/10.17504/protocols.io.bn8wmhxe). The authors confirm that all ongoing and related trials for this intervention are registered.

### Participants

Participants were recruited from 13 SMACs in Adachi City, Tokyo, Japan. The eligibility criteria were community-dwelling SMAC participants, aged 65 years old or older, at care levels between one and three who did not exercise regularly. The care level, under Japan’s LCTI, was a nationally standardised seven-level certification, which was assessed by a trained caregiver and then reviewed by the Care Needs Certification Board, a committee of medical and other professionals established at each municipality [17].⁠ Although the assessment process differed, support levels 1 and 2 were comparable to pre-frailty and care levels 1 (light) to 5 (severe) equated to frailty [18]. Care levels 4 and 5 were institutionalised or bedridden individuals, thus excluded.

Exclusion criteria included severe neuromuscular diseases, severe cerebrovascular diseases, motor disorders, severe cancer, diseases that could be affected by physical exercises, (such as myocardial infarction) or when physical exercises were prohibited for medical reasons. Also excluded were severe dementia or psychological disorders to the extent to which ARP would not be delivered. Those whose steps were not detected by the accelerometer were also excluded.

After applying the inclusion criteria, we explained the trial to the participants and obtained written consent. The allocation was made by individual at each centre and no blinding was enforced. We adopted block randomisation, using the randomizeR package for R. The block size was four.

### Intervention

ARP included weekly exercise/occupational therapy that facilitated participation with low (<3 MET) aerobic physical activity and, if possible, moderate (3 to 6 MET) physical activity (S1 Table). The participants collaborated on solving multiple tasks. To facilitate communication with local residents, the trial was conducted outside of SMACs. The frequency, intensity, type and time of physical activities are summarised in S1 Table.

In the first week, participants visited a local street to purchase the items for park cleaning and gardening. The total duration of transportation and shopping was approximately three hours. In weeks two to four, participants with and without walking aids went to a nearby park for cleaning and gardening. For cleaning, participants held a pair of tongs or a bin liner and walked around in the park to pick up litter. For gardening, they dug in the ground, planted flowers and watered them. The supervised intervention was carried out once per week, but the participants were encouraged to visit the park to take care of the flowers on other days. This four-week series was repeated three times.

While the intervention group carried out ARP, the control group remained seated and chose one of three activities from watching television, colouring in a book or folding washed clothes. The intervention was executed between December and March to minimise seasonal effects [19].⁠

### Primary and secondary outcomes

The primary outcomes of the study were daily step counts and TUG. The number of steps was counted with a tri-axis accelerometer (AM500N, ACOS Co., Ltd., Nagano, Japan) for seven consecutive days, and the mean value was adopted. Before the measurement, we manually counted the steps when the participant with accelerometer walked 100 steps to verify the accuracy of the accelerometer. The accelerometer was put on every morning by a family or professional caregiver at their home. TUG was the time required for a series of movements, in which the participant sat on a chair of 46 cm tall, stood up, walked three metres, turned around, walked back to the chair and sat [20].

Secondary outcomes include gait speed and step length in ten-metre walk test, Barthel Index and Functional Independence Measure for ADL, Mini-Mental State Examination (MMSE) for cognitive function and EuroQol 5 Diversion (EQ-5D) for QOL assessment.

### Statistical analysis

The sample size was calculated with WebPower (version 0.5.2), G*Power (version 3.1) and Power and Sample Size Calculation (version 3.1.2). We estimated a daily step count difference of 500 (standard deviation 800) and TUG difference of 1.0 (standard deviation 1.6), both of which yielded f = 0.625. Because we had two primary outcomes, alpha was adjusted in the most conservative way as 0.05/2 = 0.025 [21]. WebPower estimated n = 27 for ANOVA repeated measures between factors analysis, with the parameters ng = 2, nm = 2, nscor = 1, alpha = 0.025, beta = 0.80, f = 0.625 and type = 1 (between-group). G*Power estimated n = 22 for ANOVA repeated measures between factors analysis, with f = 0.625, alpha = 0.025, beta = 0.80, the number of groups = 2, the number of measures = 2 and correlation among repeated measures = 0.5. Power and Sample Size Calculation estimated n = 50 for Student’s *t* test, with parameters alpha = 0.025, power = 0.8 and intervention/control (m) = 1.

The differences of characteristics at baseline were compared by Student’s *t* test, Mann–Whitney’s U test or chi-squared test. For primary and secondary outcomes, jittered box-whisker figures were generated for visual inspection of normality, followed by two-way repeated measures for analysis of variance. We planned both intention-to-treat analysis and per protocol analysis.

We also conducted an ancillary analysis to compare daily step counts on a day when the participant stayed at home or went outside on his own (home), a day when the participant visited the SMAC for daycare (daycare) and a day when the participant visited the SMAC for intervention (intervention).

The statistical software used was R (version 3.6.3).

## Results

**Fig 1:**
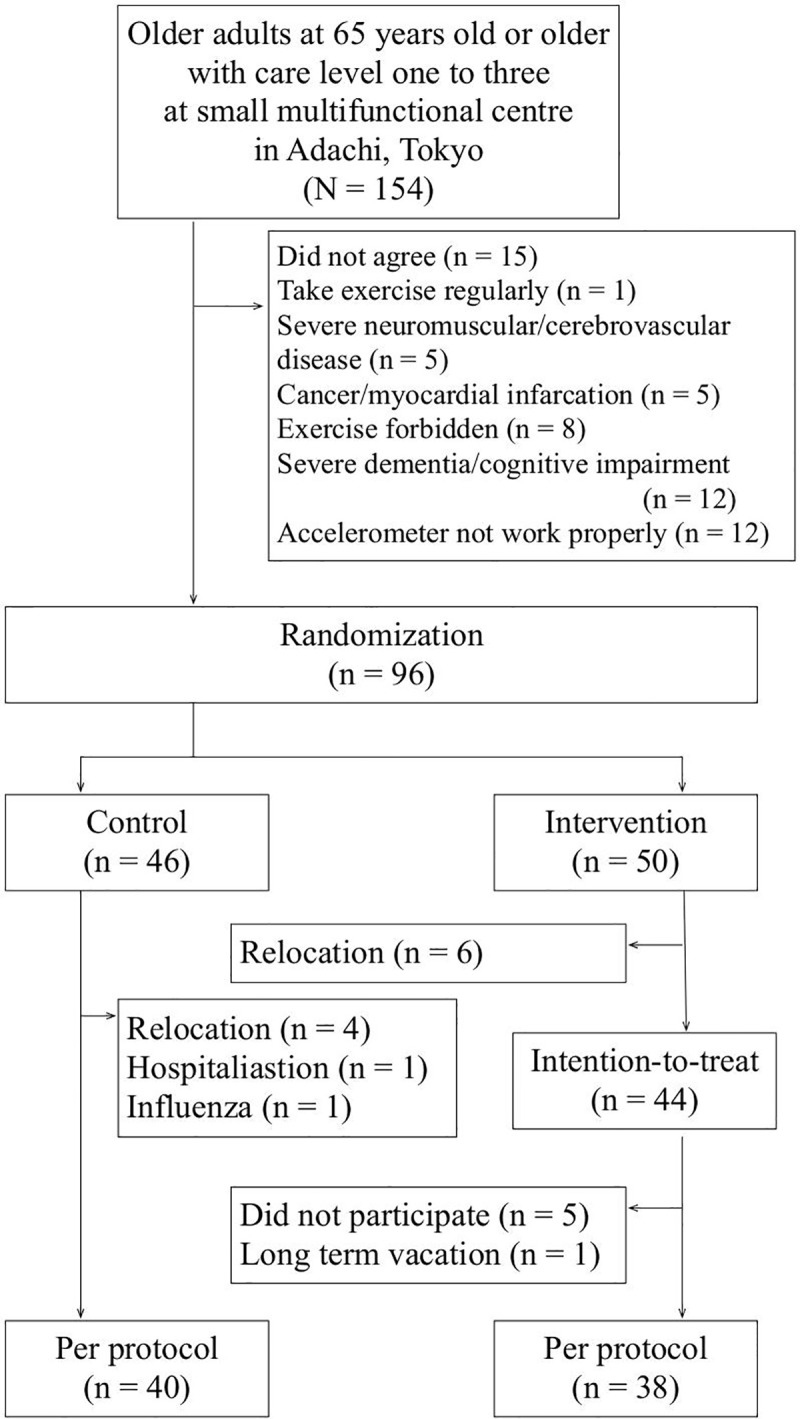
Flowchart of the study.

In December 2017, 154 clients of SMACs in Adachi, Tokyo were recruited for participation. The trial was explained, inclusion and exclusion criteria were applied and written consent was obtained from 96 participants (62%). Fifty were allocated to the intervention group and 46 to the control group (Fig 1). Due to a heavy snow on 23 January 2018, the cleaning intervention was carried out two days later than planned. No adverse event caused by the intervention was observed. The follow-up assessment was measured in March 2018.

For the intervention group, 6 participants admitted to a nursing home were lost for follow-up and 6 were excluded from per protocol analysis due to nonparticipation. The remaining 38 were included for analysis. For the control group, 4 were admitted to a nursing home, 1 was admitted to hospital and 1 with influenza (1) was lost for follow-up. The remaining 40 were included. As those who dropped out did not compete the follow-up assessment, intention-to-treat analysis was not conducted.

The results of randomisation and dropouts by facility are summarised in S2 Table.

### Baseline characteristics

There were no significant differences between the intervention group and the control group in the characteristics at baseline (Table 1). The mean age (standard deviation) was 84.1 (5.8) for intervention and 83.2 (4.3) for control. Intervention group included 26 (64.9%) females with 27 (67.5%) for control group. Care level two was most prevalent for both groups. Mean MMSE score (standard deviation) was 15.9 (4.5) for intervention and 15.0 (3.9) for control.

**Table 1:** Baseline characteristics.

	Control	Intervention	*p*
	(n = 40)	(n = 38)	
Age	83.2 ± 4.3	84.1 ± 5.8	0.45^a^
Sex			
Female	27 (67.5%)	24 (64.9%)	0.998^b^
Male	13 (32.5%)	14 (35.1%)	
Height (cm)	152.9 ± 8.5	152.7 ± 8.5	0.92^a^
Weight (kg)	49.6 ± 9.1	50.3 ± 7.2	0.69^a^
BMI (kg/m^2^)	21.2 ± 3.4	21.6 ± 2.4	0.58^a^
BIA (kg/m^2^)	10.8 ± 2.7	11.0 ± 2.2	0.69^a^
Care level			
Care level 3	9 (22.5%)	10 (26.3%)	0.50^b^
Care level 2	22 (55.0%)	16 (42.1%)	
Care level 1	9 (22.5%)	12 (31.6%)	
Daily step counts			
Mean ± SD	852 ± 394	990 ± 737	0.31^a^
Median [25%,75%]	702 [621,949]	736 [626,945]	0.75^c^
Timed Up & Go (sec)	15.9 ± 4.7	16.1 ± 5.0	0.81^a^
MMSE	15.0 ± 3.9	15.9 ± 4.5	0.35^a^

ARP: Adachi Rehabilitation Programme; BIA: Bioelectrical Impedance Analysis; BMI: Body Mass Index; MMSE: Mini-Mental State Examination.

a: Student’s *t* test

b: chi-squared test

c: Mann-Whitney’s U test.

The values are either number (percent) or mean ± standard deviation, except for the median value of daily step counts.

### Effects on physical activity and cognitive function

**Fig 2:**
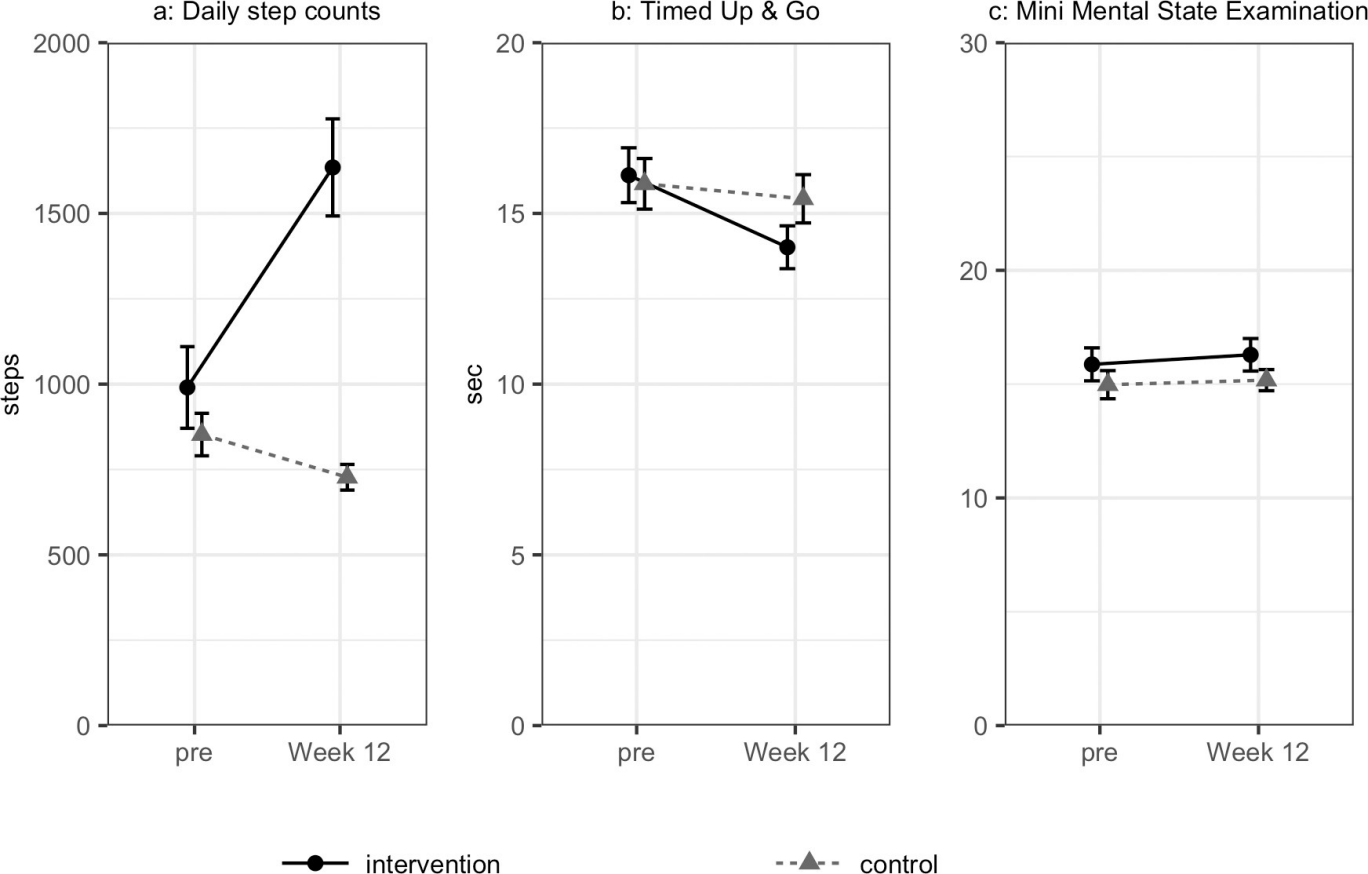
Changes in outcomes: (a) daily step counts; (b) timed up & go and (c) mini-mental state examination.

Differences in physical activity, cognitive function and health-related quality of life (HRQOL) between baseline and post-intervention are summarised in Table 2. For the control group, daily step counts decreased from 837 to 727 (*p* = 0.045), while they increased by approximately 650, from 990 to 1635, for the intervention group (*p <* 0.001) (Fig 2A). Analysis of variance showed an interaction in the intervention group (F = 14.7, *p <* 0.001). TUG decreased from 16.1 seconds to 14.0 seconds for intervention group (*p <* 0.001) without a significant interaction (Fig 2B). MMSE score increased slightly from 15.9 to 16.3 for the intervention group (*p* = 0.0095) without significant interaction (Fig 2C).

**Table 2:** Results of physical activity, performance, psychological performance and quality of life.

	Control (n = 40)				Intervention (n = 38)				Two-way repeated measures analysis of variance	
	pre-	post-	p	Difference between pre- and post-intervention (95% confidence interval)	pre-	post-	*p*	Difference between pre- and post-intervention (95% confidence interval)	F	*P*
Daily step counts (steps)	852 ± 394	727 ± 238	0.045	110.2 (–217.8, –2.6)	990 ± 737	1635 ± 877	<0.001	644.4 (460.4, 828.5)	14.68	<0.001
Timed Up & Go (seconds)	15.9 ± 4.7	15.4 ± 4.5	0.17	–0.44 (–1.08, 0.20)	16.1 ± 5.0	14.0 ± 3.9	<0.001	–2.11 (–3.22, –1.00)	1.34	0.25
10MWT										
Gait speed (m/sec)	0.82 ± 0.31	0.78 ± 0.29	0.12	–0.03 (–0.07, 0.01)	0.81 ± 0.30	0.84 ± 0.30	0.32	0.02 (–0.02, 0.06)	0.32	0.58
Step length (cm)	45.7 ± 10.9	45.2 ± 10.5	0.20	–0.56 (–1.43, 0.31)	44.8 ± 9.8	46.2 ± 10.4	0.069	1.36 (–0.11, 2.83)	0.33	0.57
Handgrip (kg)										
Overall	18.8 ± 6.0	18.0 ± 6.6	0.054	–0.74 (–1.45, 0.01)	16.6 ± 6.5	16.6 ± 6.2	0.97	–0.01 (–0.38, 0.37)	0.13	0.72
Male	24.1 ± 5.2	23.8 ± 6.1			21.0 ± 6.1	21.2 ± 5.4				
Female	16.2 ± 4.6	15.3 ± 4.8			14.0 ± 5.4	13.9 ± 5.1				
Barthel index	83.9 ± 14.9	85.0 ± 14.5	0.071	1.13 (–0.10, 2.35)	81.1 ± 18.6	81.4 ± 18.3	0.18	0.39 (–0.19, 0.98)	0.02	0.89
FIM										
Overall	88.5 ± 19.0	88.9 ± 19.1	0.41	0.45 (–0.63, 1.53)	89.7 ± 21.5	89.7 ± 21.9	0.98	–0.03 (–2.01, 1.95)	0.0053	0.94
Physical component	72.2 ± 21.4	74.8 ± 20.2	0.11	2.65 (–0.59, 5.89)	63.1 ± 21.8	66.3 ± 21.1	0.059	3.21 (–0.13, 6.55)	0.0069	0.93
Psychological component	22.6 ± 5.3	23.1 ± 4.1	0.12	0.58 (–0.16, 1.31)	21.7 ± 4.9	22.0 ± 5.0	0.14	0.26 (–0.11, 0.64)	0.040	0.84

ARP: Adachi Rehabilitation Programme; FIM: Functional Independence Measure.

The values are mean ± standard deviation.

### Steps by day

**Fig 3:**
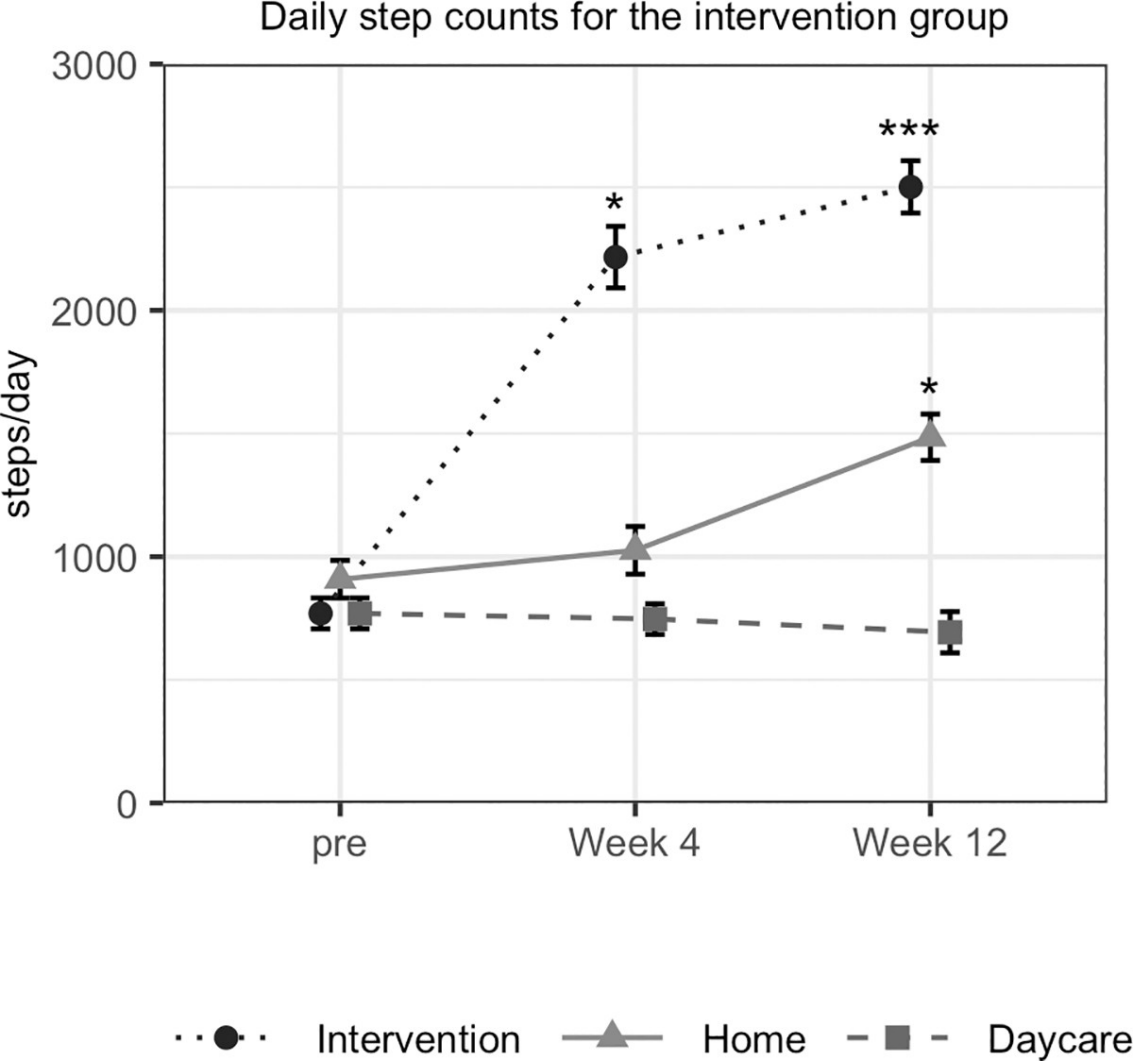
Daily step counts by day.

Daily step counts were further analysed by days for daycare, home and intervention (Fig 3, S3 Table). Counts showed a significant increase from week 4 on the intervention days (*p <* 0.001) and from week 12 on home days (*p <* 0.001), with interaction (F = 22.8, *p <* 0.001). For the control group, daily step counts on home days decreased from week 12 (*p <* 0.0076), without interaction. On home days, daily step counts of the intervention group increased significantly from 908 steps to 1485 steps, while those of the control group decreased from 865 steps to 722 steps (F = 17.1, *p <* 0.001).

Daycare was a day when participants visited daycare but did not participate in the intervention; intervention was a day when participants visited daycare and participated in the intervention; and home was a day when participants did not visit daycare.]

## Discussion

This study is the first multi-centre controlled trial to evaluate the effects of ARP on the physical activity, cognitive function and HRQOL of older adults under long-term care. Although intervention was relatively infrequent (once per week) and was supervised by professional caregivers, the intervention significantly increased daily step counts. Balance and cognitive function may have been improved, but the differences were not statistically significant.

### Effects on physical activity and cognitive function

The purpose of the study was to improve physical activity through participatory intervention. The participatory method used in ARP significantly increased daily step counts by approximately 650. Mall walking, which was a successful participatory method for healthy older adults, increased 900 steps over 8 weeks [22]. Although the frequency of supervised intervention using ARP was only once per week, the participants were encouraged to visit the park and take care of the flowers they had planted, which may have increased the step counts on other days. Meanwhile, daily step counts on daycare days did not increase, perhaps because the participants receiving daycare were sedentary and less active. It may be necessary for SMACs to implement ARP more frequently or provide other physical activity programmes besides ARP. It is important to note that the daily step counts of our trial were smaller than any other previous studies [23].

In our trial, TUG showed a tendency to improve, but it was not statistically significant. Gait speed did not show a statistically significant change. In the meta-analysis of exercise for community-dwelling older adults, there was no significant difference in TUG and gait speed [2–4,23–27]. The meta-analysis of four studies of the effects of exercise on gait speed yielded a significant difference, but only one study, which included resistance training and balance training three days per week, improved TUG and gait speed in three months [5,6]. The same group studied resistance training and functional training two days per week for patients with dementia and TUG and gait speed significantly improved [28]. Another study showed improved TUG in one year for institutionalised adults aged 75 years or older who received lower limb training while seated for one hour, three days per week [29]. Balance and ambulatory function may improve by increasing frequency and/or duration of ARP or by adding balance training at a SMAC or at home. One study in which care managers intervened every two weeks had a similar effect as supervised exercise [30]. In our trial, although ARP was explicitly included in the care plans, care managers provided monitoring once per month, which may not be sufficient.

The hypothesis of the trial did not include improvement in cognitive function and we did not find a significant increase in MMSE scores. A randomised controlled trial of 500 individuals who performed vigorous aerobic exercise and strength training for 60 minutes twice per week did not find a delay the development of cognitive impairments [31]. Meanwhile, procedural memory may have been preserved for individuals with dementia in a study where a 90-minute exercise session once per week improved MMSE in 40 weeks [23]. Another study included a mixture of cognitive training and exercise for 60 minutes, 5 days per week. Individuals in that study showed a slight improvement in 6 months in ADAS-cog, but not in MMSE [32]. It may be more important to note that professional caregivers reported behavioural and psychological symptoms of dementia, such as insomnia and incontinence, were alleviated on intervention days. This needs to be studied in comparison to other psychosocial behaviour management programmes [33].

### Limitations

This study has several limitations. First, the number of participants was not large enough for randomisation at some facilities, hence the study design had to be modified to be a quasi-randomised trial. SMACs, by legislation, have small capacities, up to 18 patients at each site per day or less depending on the floor area of the facility. We conducted our trial at 11 SMACs, some of which had a small sample size, resulting in poor randomisation. Some have, however, argued that smaller facility capacity might be associated with better dementia care or better quality of environment [34,35]. Thus, a further study of appropriate randomisation method including small capacity facilities will be needed. Second, although our trial met sample size estimations for two-way repeated measures ANOVA, our results did not show a significant interaction in TUG despite that TUG seems to have significantly decreased for the control. This may have been due to inappropriate parameters, e.g. effect size or correlation, when we estimated the sample size [36].Third, intention-to-treat analysis was not conducted as some of the participants who dropped did not visit the facility on the assessment day. We cannot exclude the possibility that those who were vulnerable to physical improvement may have dropped out. Fourth, we conducted two distinct practices, i.e. shopping and gardening and cannot distinguish the effectiveness of each separate practice. Lastly, although the dropout rate was relatively small compared to other trials [37], we did not determine to what extent shopping/gardening contributed to high adherence rate. We were unable to follow-up with participants at 12 months after the intervention, because more than one-half of our participants were not available at follow-up.

### Clinical implications

It may be difficult for older adults under long-term care to walk outdoors on their own. ARP is a cost-effective and safe method and may have effectively increased the physical activity of older adults under long-term care by increasing motivation and changing behaviour.

Currently, there is no consensus on the minimum clinically important differences in daily step counts, but research estimated 600 steps for step counts. ARP may have successfully achieved the minimum clinically important differences in daily step counts.

## Supporting information

S1 Checklist TREND statement checklist. (DOC)

S1 Table Basic components of the Adachi Rehabilitation programme. Note: The unit of time is minutes. The basic component is a four-week programme. The programme was repeated three times (total of 12 weeks).
(DOC)

S2 Table Results of randomisation and dropouts by facility. (DOC)

S3 Table Daily step counts of the intervention group by day. ARP: Adachi Rehabilitation Programme. The values at pre-intervention, Week 4 and Week 12 are steps/day (mean ± standard deviation).
(DOC)

S1 File (PDF)

S2 File (PDF)

S1 Data (GZ)
