# Supplementary material for: Effects of Adachi Rehabilitation Programme on older adults under long-term care: A multi-centre controlled trial
Source: PLoS One. 2021 Feb 12;16(2):e0245646. doi: 10.1371/journal.pone.0245646 (PMC7880461; doi:10.1371/journal.pone.0245646)
Supplement: S1 Table — Note: The unit of time is minutes. The basic component is a four-week programme. The programme was repeated three times (total of 12 weeks). (DOC) [file pone.0245646.s002.doc]

S1 Table. Basic component of Adachi Rehabilitation Programme

| Frequency | Intensity | Type | Time |
| --- | --- | --- | --- |
| Once a week | low to moderate | Walk to bus stop | 10 |
| (Week 1) | low | Bus ride | 30 |
|  | low | Stroll in the shopping street | 60 |
|  |  | Rest | 10 |
|  | low | Bus ride | 30 |
|  | low to moderate | Walk from bus stop | 10 |
|  |  | Total | 150 |
| Once a week | low to moderate | Walk to park | 5 |
| (Weeks 2 to 4) | moderate | Plant flowers | 10 |
|  | low | Pick up garbages | 40 |
|  | low to moderate | Walk from park | 5 |
|  |  | Total | 60 |

Note: The unit of time is minutes.

The basic component is a four-week programme. The basic component was repeated three times (12 weeks).
