# Supplementary material for: Effects of Adachi Rehabilitation Programme on older adults under long-term care: A multi-centre controlled trial
Source: PLoS One. 2021 Feb 12;16(2):e0245646. doi: 10.1371/journal.pone.0245646 (PMC7880461; doi:10.1371/journal.pone.0245646)
Supplement: S2 Table — (DOC) [file pone.0245646.s003.doc]

S2 Table. Results of randomisation and dropouts by facility

| Facility | Control | |  | Intervention | | |
| --- | --- | --- | --- | --- | --- | --- |
|  | Allocation | Dropout |  | Allocation | Dropout | Excluded  for per protocol |
| 1 | 10 | 2 |  | 11 | 3 | 3 |
| 2 | 5 |  |  | 7 | 1 | 1 |
| 3 | 5 | 3 |  | 6 | 1 |  |
| 4 | 3 |  |  | 3 |  | 1 |
| 5 | 7 |  |  | 5 | 1 | 1 |
| 6 | 2 |  |  | 2 |  |  |
| 7 | 2 |  |  | 1 |  |  |
| 8 | 2 |  |  | 1 |  |  |
| 9 | 5 | 1 |  | 8 | 1 |  |
| 10 | 3 |  |  | 3 |  |  |
| 11 | 1 |  |  | 3 |  |  |
| 12 |  |  |  |  |  |  |
| 13 |  |  |  |  |  |  |
| Total | 46 | 6 |  | 50 | 6 | 6 |
