# Supplementary material for: Effects of Adachi Rehabilitation Programme on older adults under long-term care: A multi-centre controlled trial
Source: PLoS One. 2021 Feb 12;16(2):e0245646. doi: 10.1371/journal.pone.0245646 (PMC7880461; doi:10.1371/journal.pone.0245646)
Supplement: S3 Table — ARP: Adachi Rehabilitation Programme. The values at pre-intervention, Week 4 and Week 12 are steps/day (mean ± standard deviation). (DOC) [file pone.0245646.s004.doc]

S3 Table. Daily step counts of the intervention group by day

|  | The number of days measured | pre-intervention | Week 4 | Week 12 | Two-way repeated analysis of variance | |
| --- | --- | --- | --- | --- | --- | --- |
|  |  |  |  |  | F | p |
| Day of daycare with intervention |  |  |  |  |  |  |
| Intervention | 1 |  | 2217 ± 770 | 2501 ± 655 |  |  |
|  |  |  |  |  |  |  |
| Day of daycare (without intervention) |  |  |  |  |  |  |
| Control | 3 | 737 ± 691 | 818 ± 704 | 730 ± 357 | 0.23 | 0.80 |
| Intervention | 3 (pre-intervention)  or 4 (Weeks 4 and 12) | 770 ± 649 | 747 ± 511 | 693 ± 393 |  |  |
|  |  |  |  |  |  |  |
| Day of staying at home |  |  |  |  |  |  |
| Control | 3 | 865 ± 729 | 841 ± 895 | 722 ± 363 | 17.1 | <0.001 |
| Intervention | 3 | 908 ± 958 | 1026 ± 1217 | 1485 ± 1184 |  |  |

ARP: Adachi Rehabilitation Programme
