# Supplementary material for: Effects of Adachi Rehabilitation Programme on older adults under long-term care: A multi-centre controlled trial
Source: PLoS One. 2021 Feb 12;16(2):e0245646. doi: 10.1371/journal.pone.0245646 (PMC7880461; doi:10.1371/journal.pone.0245646)
Supplement: S1 File — (PDF) [file pone.0245646.s005.pdf]

第 II 種

要介護高齢者を対象とした参加を促進する地域リハビリテーションの開発とその効用検証

臨床試験登録番号: UMIN-CTR UMIN000028317

研究責任医師
教授　上月　正博
東北大学大学院医学系研究科内部障害学分野
〒980-8574　仙台市青葉区星陵町 1-1
TEL　022-717-7351　FAX 022-717-7355
E-mail: kohzuki@med.tohoku.ac.jp
研究事務局
馬場　美彦
東北大学大学院医学系研究科内部障害学分野
〒980-8574　仙台市青葉区星陵町 1-1
TEL　022-717-7353　FAX 022-717-7355
E-mail: babayoshihiko@mac.com

目次

1 目的 6

2 背景と研究計画の根拠 6
2.1 背景 6
2.2 研究の科学的合理性の根拠 7

3 研究対象者の選定方針 7
3.1 適格基準 7
3.2 除外基準 7

4 研究の方法、期間 8
4.1 研究デザイン 8
4.2 治療・介入計画の内容 8
4.3 併用療法 9
4.4 後治療 9
4.5 検査スケジュール 9
4.6 研究期間 9

5 有害事象の評価 10
5.1 情報の入手 10
5.2 有害事象の記載 11

6 重篤な有害事象／不具合発生時の対応（研究機関の長に報告する有害事象範囲を含む） 12
6.1 有害事象／不具合発生時の対応 12
6.2 研究機関の長、研究責任医師（研究代表者）への報告 12
6.3 厚生労働大臣（行政当局）への報告 12
6.4 効果安全性評価委員会への対応 13
6.5 情報の公開 13

7 調査項目・方法 13
7.1 主要評価項目 13
7.2 副次的評価項目 13
7.3 評価の中央判定 14

8 登録・割付 14
8.1 登録 14
8.2 割付 15

9 予定症例数、設定根拠 15
9.1 予定症例数 15
9.2 設定根拠 15
9.3 研究対象者登録見込み 15

10 統計解析 15
10.1 統計解析の方法 15
10.2 中間解析と研究の早期中止 15

11 データの管理方法、自己点検の方法 15
11.1 症例記録（Case Report Form：CRF）の作成 15

11.2 CRF の自己点検 16
11.3 CRF の送付及び保管 16
11.4 CRF の修正手順 16

12 インフォームド・コンセントを受ける手続 16
12.1 研究対象者への説明 16
12.2 同意 17

13 代諾者等からインフォームド・コンセントを受ける場合の手続 17

14 インフォームド・アセントを得る場合の手続 17

15 個人情報等の取扱い 17
15.1 個人情報の利用目的 17
15.2 利用方法（匿名化の方法） 17
15.3 安全管理責任体制（個人情報の安全管理措置） 17

16 研究対象者に生じる負担、予測されるリスク（起こりうる有害事象を含む）・利益、これらの総合的評価、負担・リスクを最小化する対策 17
16.1 研究参加に伴って予測される利益と不利益の要約 17

17 試料・情報等の保存・廃棄の方法 18
17.1 保存 18
17.2 廃棄 18

18 研究の資金源、研究機関の研究に係る利益相反及び個人の収益等、研究者等の研究に関する利益相反に関する状況 18

19 知的財産 18

20 研究に関する情報公開の方法 18
20.1 研究計画の登録 18
20.2 研究結果の登録 19
20.3 研究結果の公表 19

21 研究機関の長への報告内容、方法 19

22 研究対象者等、その関係者からの相談等への対応 19

23 緊急状況下に研究を実施する場合、要件全ての充足を判断する方法 19

24 研究対象者等に経済的負担または謝礼がある場合、その旨、その内容 19

25 侵襲を伴う研究の場合、研究によって生じた健康被害に対する補償の有無、内容 20

26 業務内容、委託先の監督方法 20

27 試料・情報が同意を受ける時点では特定されない将来の研究のために用いられる可能性／他の研究機関に提供する可能性がある場合、その旨と同意を受ける時点において想定される内容 20

28 モニタリングの実施体制 20

29 研究計画書の変更 21

30 研究の実施体制 21
30.1 研究機関の名称、研究責任医師の氏名 21
30.2 共同研究機関 21
30.3 研究事務局、共同研究機関、研究責任者の役割・責任 21
30.4 効果安全性評価委員会 22
30.5 統計解析、データセンター 22
30.6 研究に関する問合せ窓口 22

31 引用文献 23

32 Appendix 25

概要

シェーマ

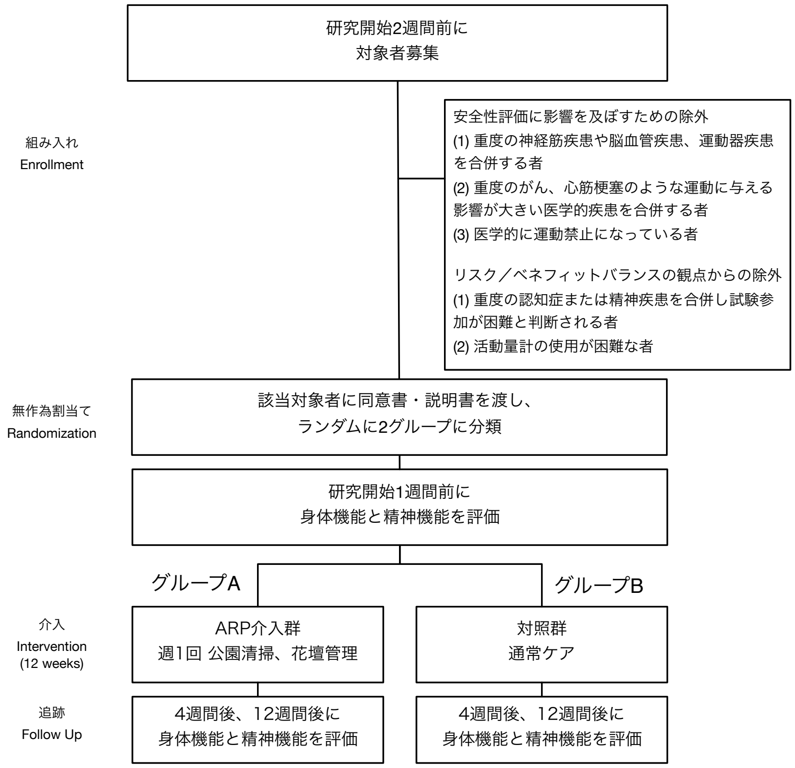

目的

介護保険サービスを利用する高齢者を対象に、参加を促進する地域リハビリテーションとして足立リハビリテーションプログラム (Adachi Rehabilitation Programme, 以下 ARP) を開発し、ARP の活動と心身機能に対する効果を、通常ケアとのランダム化比較検証することとする。

対象

東京都足立区の小規模多機能型居宅介護施設を利用している要介護 1〜3 の高齢者。

治療

ARP は、週 1 回の参加、4 週間を 1 サイクルとする地域リハビリテーションプログラムである。

第 1 週は、週に 1 回、バスに約 10〜20 分乗って、公園清掃と花壇管理に必要な物を買いに行く。移動と買い物にかかる時間は 3 時間である。次の 3 週間は、週に 1 回 1 時間、近所の公園で清掃活動と花壇管理を行う。公園は、介護事業所の近隣 (500m 以下) にあり、面積は約 1,000m^2 で多少の起伏がある。公園まで徒歩 (杖、シルバーカーあり) で行く。清掃活動は、トングとゴミ袋を持ち、公園内を自由に移動してゴミを拾う。花壇管理は、土を掘り、花の株を植え、ホースで水遣りを行う。これらは、足立区の「公園の自主管理」と「花壇の自主管理」制度を利用している。

これを 3 サイクル (12 週間) 行う。その他の日は、通常通りに生活する。

グループA　週 1 回、上記 ARP を行う。

グループB　介入群が ARP を行っている間、通常ケアとして、主に座位でテレビ鑑賞、塗り絵、洗濯物たたみの 3 種類の

活動から選んでいただく。

予定症例数、研究期間

(1) 予定症例数: 歩数、Timed Up and Go における有為な改善が出現し、有意水準 ($\alpha = 0.05$) および効果量 ($\delta = 0.50$)、検定力 ($1 - \beta = 0.80$) として、対応あり t 検定で両側検定を実施すると仮定した場合、各グループの対象者は 50 名、合計 100 名が必要である。

グループ A が 50 名、グループ B が 50 名であり、グループ A は ARP 介入群、グループ B は対照群とする。脱落が 1 割程度と想定し、120 名 (グループ A 60 名、グループ B 60 名）の登録を見込んでいる。

(2) 研究期間: 西暦2017年11月 (倫理委員会承認後) 〜 西暦 2021 年 9 月

問合せ先

馬場　美彦

東北大学大学院医学系研究科内部障害学分野
〒980-8574　宮城県仙台市青葉区星陵町 1-1
TEL : 022-717-7353 FAX : 022-717-7355

小規模多機能型居宅介護　じゃすみん扇
〒123-0873 東京都足立区扇 1-31-32
TEL: 03-6807-1278 FAX: 03-6807-1279

E-mail: babayoshihiko@mac.com

1　目的

要介護 1〜3 の高齢者を対象とした参加を促進する足立リハビリテーションプログラム (ARP) が、対象者の意欲が向上し、日常の身体活動量が増加し、またバランス能などの身体機能が改善するという有効性を、通常ケアとのランダム化比較にて評価する。

主要評価項目は歩数と Timed Up and Go である。

2　背景と研究計画の根拠

2.1　背景

本邦では、要介護高齢者の自立支援を目的に 2000 年に介護保険制度が発足し、より低価格で多くのサービスを提供できるようになったことが評価されている [1] [2] 。しかしながら、在宅介護の質に課題を残しているなどの指摘もなされている [2] 。さらに、高齢者の生活期リハビリテーションは、医療保険から介護保険に移行することが検討されているが、急性期・回復期リハビリテーションと比較して心身機能・身体構造の改善が難しいという課題がある。そこで厚生労働省では、「心身機能・身体構造」の改善だけでなく、残存能力を活用した買い物や外出などの「活動」「参加」をリハビリテーションとして勧めている [3]。

居宅サービス計画（ケアプラン）では、調理などの日常生活における「活動」や、近隣の公園への散歩などの「参加」、商店街での買い物、公共交通機関の利用などの「参加」の機会を提供している。国際生活機能分類 (International Classiffication of Functioning, Disability and Health; 以下、ICF) 以前は、「歩くことができるようになったら外出する」というように、心身機能を改善することがリハビリテーションと捉えられていたが、ICF では、「参加」が逆に「活動」を改善する可能性が示唆されている [4]。このことは、健常高齢者を対象とした大規模な観察研究でも確かめられている [5] [6]。「活動」が低下した高齢者に対し「参加」を促進することは、日常の身体活動量の増加やバランス能力など「活動」にも改善効果があると仮定されるが、本研究のように週 1 回という少ない介入で効果を検証した介入研究はない。

以上のような背景から、介護現場で行われているプログラムにはリハビリテーションとしての効果があると予想されるが、まだ評価されていない。そこで本研究では、足立区の介護事業所で行われている参加プログラムを、足立リハビリテーションプログラム (Adachi Rehabilitation Programme, 以下 ARP) と命名し、要介護状態の高齢者に対して、ARP の「活動」「心身機能」への効果を明らかにするためにランダム化比較試験を行う。

ARP とは、介護保険サービスを利用している要介護高齢者を対象に、公園において清掃活動と花壇管理を行うことと、公

共交通機関を利用して買い物に出かけることを介入として、ARP の身体活動量、バランス能力、認知機能、QOL などの心身機能への効果を評価する。

研究実施施設は、研究分担者（馬場美彦）が世話人を務める足立区多機能サービス連絡会の会員事業所とする。足立区多機能サービスは、足立区内のすべての小規模多機能型居宅介護 (13 事業所) によって構成されている。

2.2　研究の科学的合理性の根拠

介護サービスで行なわれている高齢者の機能訓練を生活期リハビリテーションと捉えるには、幾つかの課題があると考えられる [7] [8] [9] [10] [11]。

まず、ケアマネージメントにリハビリテーションを組み込む必要があるが、介護保険で作成される居宅サービス計画（ケアプラン）には、訪問リハビリテーションの利用率は要介護 4 で半数、要介護 5 が 17% で、要介護 3 以下ではほとんど利用されていない [7]。訪問リハビリテーションは、介護保険において最も使われていないサービスであり [8]、介護支援専門員 (ケアマネージャー) に啓発が必要であるとの指摘もある [7] [8] [9] 。

次に、自宅や地域でリハビリテーションを行うには、転倒などのリスクを軽減する環境の設定が重要になる [12]。介護施設内での環境整備については研究があるものの [13]、要介護者が外出するために必要な環境整備や、要介護者の外出が心身機能へどのような影響を与えるかを調査した研究はない。高齢者施設でさえも転倒が多く [10]、自宅や地域でリハビリテーションを行うことは、転倒のリスクが伴う [14]。

さらに、介護におけるリハビリテーションのシステマティックレビューによると、49 の研究のうち 30 が米国であり、また対象者を施設在住であることを検索条件にしている [15]。これは、日本における在宅での介護とは環境が異なることを示唆している。国内の介護保険サービス利用者についての研究は、脳卒中患者を対象にしたものなどに限られ、日常生活動作 (Activities of Daily Living, ADL) 改善に効果は認められているが、十分な科学的根拠が得られていないと論じている [8]。退院時においては、リハビリ専門職と介護職の間でケアカンファレンスを行っているが、退院後のリハ継続やそのアウトカムについてはほとんど調査がないという報告もある [11]。

以上から、ケアプランに位置付けることのできる、介護保険サービスで転倒などのリスクの少ない状態で行えるプログラムを開発し、その心身機能や QOL への影響を調査することは、高齢者の地域におけるリハビリテーションに示唆を与えるものと考えられる。

本研究で開発した ARP を行うことで得られる費用対効果は、医療技術評価 (Health Technology Assessment, HTA) の手法で、研究責任医師および研究実施者が包括的に判断する。そして、リスクを最小化し、費用対効果を改善するさらなる措置を検討することとする。

本研究を実施することの適否については、倫理的、科学的および医学的妥当性の観点にもとづいて、東北大学病院臨床研究倫理委員会が審査し、研究機関の長による承認を得るものとする。

3　研究対象者の選定方針

3.1　適格基準

(1) 要介護度 1～3
(2) 定期的な有酸素運動又は筋力トレーニングを行っていない者
(3) 研究の説明を実施し、研究への参加同意を書面で得た者
(4) 年齢: 65 歳以上
(5) 性別: 男女
(6) 地域在住で、東京都足立区の小規模多機能型居宅介護施設の利用者
(7) 報償費を受け取っていない利用者

3.2　除外基準

■（安全性評価に影響を及ぼすための除外）
(1) 重度の神経筋疾患や脳血管疾患、運動器疾患を合併する者
(2) 重度のがん、心筋梗塞のような運動に与える影響が大きい医学的疾患を合併する者
(3) 医学的に運動禁止になっている者

■（リスク／ベネフィットバランスの観点からの除外）

(1) 重度の認知症または精神疾患を合併し試験参加が困難と判断される者

(2) 活動量計の使用が困難な者

重度の認知症または重度の精神疾患とは、認知症または精神疾患のために ARP または検査項目を実行できない、あるいは認知症状または精神疾患が悪化する恐れがあると医師が判断した時に除外とする。この際、後述する副次的評価項目の一つである MMSE を使用する。なお、この後 2 週間以内に ARP を開始する場合は、この際の MMSE 値をベースライン（０ヶ月）の MMSE 値とする。

4 研究の方法、期間

4.1 研究デザイン

(1) 研究デザイン

対照: ARP 対照 (ARP 介入群 / 対照群)

盲検化: 非盲検化

比較方法: ランダム化並行群間比較, 研究対象者内前後比較

(2) 研究デザインの設定根拠

ARP の優越性を示すための試験である。

4.2 治療・介入計画の内容

ARP の実施許可を得た小規模多機能型介護施設を利用する対象者に対し、研究の説明を行い同意して頂けるか確認する。同意を得られた全ての対象者に介入 2 週間前まで、適格基準と除外基準を説明し、それぞれの基準に該当しているのかを確認する。対象者をランダムにグループ A とグループ B に分類する。

足立区の小規模多機能型居宅介護

1	NPO くまハウス	こぐま	鹿浜 3-17-23	03-3854-4121
2	(福) すこやか福祉会	よりみちの家	柳原 1-29-16	03-5284-2165
3	(株) ケアサービスとも	ともの家	花畑 1-23-13	03-5831-6645
4	(有) アウトソー	じゃすみんの家	西新井 7-10-13	03-5647-9111
5	(株) ヒューマンサービス	ほほえみのいえ	伊興 1-6-22	03-5838-2797
6	NPO ぷらちなくらぶ	スマイルぷらちな	綾瀬 7-4-5	03-5856-1850
7	ミモザ (株)	ミモザ千住桜木	千住桜木 2-14-10	03-5284-3828
8	(有) アウトソー	じゃすみん扇	扇 1-31-32	03-6807-1278
9	(株) ケアサービスとも	あおいの家	青井 2-16-8 1F	03-6807-2613
10	(有) アウトソー	じゃすみん花畑	花畑 3-5-17	03-5856-7032
11	(株) コンフォート	コンフォートエルバ綾瀬	綾瀬 2-14-14	03-5629-3080
12	(株) 大熊	小規模多機能ホームしまなみ	椿 2-22-2	03-5856-9537
13	医療法人社団 苑田会	ナーシングホームそのだ	保木間 1-29-12	03-5831-0400

研究分担者の馬場美彦は、じゃすみんの家、じゃすみん扇、じゃすみん花畑のホーム長であり、また上記 13 事業所から構成される足立区多機能サービス連絡会の世話人として、各事業所の管理者・計画作成担当者と研究の実施に関する連絡調整等を行う。

ARP は、週 1 回の参加、4 週間を 1 サイクルとする地域リハビリテーションプログラムである。

第 1 週は、週に 1 回、バスに約 10〜20 分乗って、公園清掃と花壇管理に必要な物を買いに行く。移動と買い物にかかる時間は 3 時間である。次の 3 週間は、週に 1 回 1 時間、近所の公園で清掃活動と花壇管理を行う。公園は、介護事業所の近隣 (500m 以下) にあり、面積は約 1,000m^2 で多少の起伏がある。公園まで徒歩 (杖、シルバーカーあり) で行く。清掃活動は、トングとゴミ袋を持ち、公園内を自由に移動してゴミを拾う。花壇管理は、土を掘り、花の株を植え、ホースで水遣り

を行う。これらは、足立区の「公園の自主管理」と「花壇の自主管理」制度を利用している。
これを 3 サイクル (12 週間) 行う。その他の日は、通常通りに生活する。

グループA 週 1 回、上記 ARP を行う。
グループB 介入群が ARP を行っている間、通常ケアとして、主に座位でテレビ鑑賞、塗り絵、洗濯物たたみの 3 種類の活動から選んでいただく。

分類された各々の 2 グループに対して、研究実施施設にて身体機能と精神機能の評価を実施する。有効性の主要評価項目として 1 週間の活動量 (歩数) と TUG (Timed Up and Go) を評価する。副次的評価項目としては、BI (Barthel Index)、FIM (Functional Independence Measure) 、MMSE (Mini Mental State Examination)、1 週間の活動量 (MET・時)、10m 最大歩行、握力を評価する。その後、グループ A に ARP を、グループ B には通常ケアを 12 週間実施する。4 週間後、12 週間後に身体機能と精神機能の評価を実施する。
本プログラムを含めすべての評価は、研究責任医師あるいは研究分担者が行う。
(1) 試験機器
歩数・活動量の計測には、ACOS 社の歩数計 AM500N を使用する。歩数計は研究室で所有しているものを貸与し、7 日間連続で装着する。
握力の計測には、スメドレー式握力計 竹井機器工業 T.K.K.5401 グリップ-D デジタル握力計を使用する。
(2) 介入レベル
対象者に対し、研究の説明を行い同意して頂けるか確認する。同意を得られれば、対象者に運動介入 2 週間前まで、適格基準と除外基準を説明し、それぞれの基準に該当しているのかを確認する。
(3) 介入レベルの変更基準
本研究においては、全ての介入における内容・用量は研究が終了するまで変更することはない。研究対象者が急務により ARP を 1 回以上休み、全ての介入が実施できない場合でも次の介入は規定とおり進む。万が一、ARP が持続困難な場合、該当対象者を脱落として見なす。
(4) 中止・完了基準
運動実施中に、研究対象者に対し、重篤な有害事象又は有害事象による研究拒否もしくは転居や介護保険サービスの変更などを含む有害事象に関連しないその他の理由により研究拒否した場合、すぐに介入を中止する。また、研究対象者が有害事象の発現なしに ARP 介入を終えた場合、介入完了と見なす。
(5) 個人情報
本研究に関する個人情報の管理と匿名化は、足立区多機能サービスが行う。
個人情報管理責任者 馬場 美彦 （足立区多機能サービス連絡会 世話人）

4.3 併用療法

(1) 許容する併用療法: 試験前から使用している薬や栄養療法。散歩や階段昇降など、日常生活に必要な有酸素性運動。
(2) 許容されない併用療法: ARP 以外の有酸素性運動、筋力トレーニング

4.4 後治療

ARP 中止後には、ARP は行わない。
ARP 終了後は、引き続き ARP を実施する。

4.5 検査スケジュール

研究前、研究 4 週間後、研究 12 週間後に検査を行う。

4.6 研究期間

研究期間: 西暦2017年 11月（倫理委員会承認後）～ 西暦 2021 年 9 月
対象者の参加期間: 西暦 2017年11月（倫理委員会承認後）～ 西暦 2018 年 2 月

月	週	グループ A	グループ B
		ARP 介入群	対照群
11 月	4	身体機能・精神機能の評価 (研究前)	
12 月〜2 月	1	バス乗車、買物	事業所内で通常ケア
	2	公園での清掃	事業所内で通常ケア
	3	公園での清掃	事業所内で通常ケア
	4	公園での清掃	事業所内で通常ケア
3 月	1	身体機能・精神機能の評価 (12 週間後)	

「事業所内で通常ケア」とは、ARP 介入群が ARP を行っている間、主に座位でテレビ鑑賞、塗り絵、洗濯物たたみの 3 種類の活動から選んでいただく。

5 有害事象の評価

有害事象とは、研究を実施した研究対象者および術者等に生じたすべての好ましくない、または意図しない疾病または障害並びにその徴候（臨床検査値の異常を含む）をいい、試験薬/試験機器との因果関係の有無は問わない。

有害事象の収集は、研究実施以降、個々の研究対象者の観察期間終了または中止時までの期間とする。

5.1 情報の入手

(1) 研究者等（担当医）は、重篤な有害事象／不具合が発現した場合、適切な処置を行い、研究機関の研究責任者に報告する。

(2) 研究機関の研究責任者は、研究者等に以下を確認する。

研究責任者による研究者等への確認事項

1. 有害事象名／不具合名
2. 重症度分類 1)
3. 重篤性 2) 、重篤と判断した理由
4. 予測性（未知・既知）3)
5. 介入（試験薬／試験機器）との因果関係
6. 事象／不具合の経緯（発現日、経過、転帰等）
7. 被験者の特定に関する情報（イニシャル、年齢、性別）

1) 重症度分類

National Cancer Institute Common Terminology Criteria for Adverse Events
(NCI CTCAE v4.0 : http://www.jcog.jp/doctor/tool/CTCAEv4J_20150310.pdf)
等に従って判定する。

NCI CTCAE 分類に該当する項目がない場合、以下「有害事象の重症度分類基準」を参考に判定する。

重症度分類（NCI CTCAE Grade）	基準
軽症 （Grade1）	症状がない、または軽度の症状がある。臨床所見または検査所見のみ。治療を要さない。
中等症 （Grade2）	最小限/局所的/非侵襲的治療を要する。年齢相応の身の回り以外の日常生活動作の制限*。
重症 （Grade3）	重症または医学的に重要であるが、ただちに生命を脅かすものではない。入院または入院期間の延長を要する。活動不能/動作不能。身の回りの日常生活動作の制限**。
最重症 （Grade4）	生命を脅かす。緊急の処置を要する。
死亡 （Grade5）	有害事象（AE）による死亡。

*身の回り以外の日常生活動作（instrumental ADL）
：食事の準備、日用品や衣類の買い物、電話の使用、金銭の管理等。
**身の回りの日常生活動作（self care ADL）
：入浴、着衣・脱衣、食事の摂取、トイレの使用、薬の服薬が可能で、寝たきりではない状態。

2) 重篤の定義

研究責任者による研究者等への確認事項

- 死に至るもの
- 生命を脅かすもの
- 治療のための入院又は入院期間の延長が必要となるもの
- 永続的又は顕著な障害・機能不全に陥るもの
- 子孫に先天異常を来すもの

研究計画書で規定する入院、研究前（同意取得前）より予定していた療法または検査を研究実施中に実施することのみを目的とした入院（予定手術や検査等）、有害事象に伴う治療・検査の目的以外の入院（健康診断等）は重篤な有害事象として取扱わない。

3) 予測性の定義

研究責任者による研究者等への確認事項

〇予測できない（未知）
当該事象等の発現、あるいは発現数、発現頻度、発現条件等の発現傾向が当該試験薬／試験機器に関する公式文書（添付文書や論文等）から予測できないもの
〇予測できる（既知）
当該事象等の発現、あるいは発現数、発現頻度、発現条件等の発現傾向が当該試験薬／試験機器に関する公式文書（同上）から予測できるもの

5.2　有害事象の記載

研究者等は、発現したすべての有害事象に関し、有害事象名、程度（重篤、非重篤）、重篤と判断した理由、発現日、転帰日、処置、転帰（回復、軽快、回復したが後遺症あり、未回復、死亡）、研究内容との因果関係、コメント（因果関係と判定理由等）を症例報告書に記載する。

本研究は、屋外での活動を含むために健康被害が生じる恐れがある。介入日には、体温、血圧を事前に計測し、試験実施施設の医師・看護師が参加の可否を判断する。

循環動態の変動に伴うめまい、顔色不良、多量発汗、息切れ、血圧や心拍数の異常などは現れる可能性が考えられる。また、下肢の関節痛や筋肉痛の運動器障害が生じる可能性がある。有害事象が発生した場合には、医師や看護師が適切に対応する。

6　重篤な有害事象／不具合発生時の対応（研究機関の長に報告する有害事象範囲を含む）

6.1　有害事象／不具合発生時の対応

(1) 研究者等は、有害事象／不具合が発現した場合、適切な処置を施し、研究対象者の安全確保に留意して原因究明に努める。

(2) 研究者等は、発現した症状あるいは臨床検査値の異常変動について、原則として当該事象が消失または研究開始前の状態に回復するまで、または臨床上問題とならないと判断されるまで、可能な限り経過観察を継続し、その転帰を確認する。

(3) 研究終了時に未回復の有害事象／不具合が非可逆的な事象の場合等、研究者等が追跡不要と判断した場合、研究対象者の研究終了時をもって追跡終了し、症例報告書のコメント欄に追跡不要と判断した理由を記載する。

6.2　研究機関の長、研究責任医師（研究代表者）への報告

(1) 研究機関の研究責任者は、重篤な有害事象／不具合の発現を知った時点から以下の期限内に研究機関の長に報告する。報告は、「(様式第 9 号) 重篤な有害事象に関する報告書」*を用いる。

*臨床研究に関する様式ダウンロード `http://www.med.tohoku.ac.jp/public/rinri_d.html`

<table>
<tr><td rowspan="3"></td><td colspan="3">軽症/中等症/重症 (Grade 1/2/3)</td><td colspan="2">最重症 (Grade 4)</td><td colspan="2">死亡</td><td rowspan="3">その他
医学的に
重要な
状態</td></tr>
<tr><td>予測できる
(既知)</td><td colspan="2">予測できない
(未知)</td><td rowspan="2">予測できる
(既知)</td><td rowspan="2">予測できない
(未知)</td><td rowspan="2">予測できる
(既知)</td><td rowspan="2">予測できない
(未知)</td></tr>
<tr><td>入院
なし/あり</td><td>入院
なし</td><td>入院
あり</td></tr>
<tr><td rowspan="2">因果関係あり</td><td rowspan="2">報告
不要</td><td rowspan="2">報告
不要</td><td>初回報告
：10 日以内
追加報告
：随時</td><td colspan="4">一時報告：72 時間以内
二次報告: 7 日以内
追加報告: 随時</td><td rowspan="2"></td></tr>
<tr><td>厚労大臣
報告対象
Grade 3 のみ</td><td></td><td>厚労大臣
報告対象</td><td></td><td>厚労大臣
報告対象</td></tr>
<tr><td>因果関係なし</td><td>報告
不要</td><td>報告
不要</td><td>初回報告
：10 日以内
追加報告
：随時</td><td colspan="4">一時報告：72 時間以内
二次報告: 7 日以内
追加報告: 随時</td><td></td></tr>
</table>

*介入中または最終プロトコール介入日から 30 日以内のみ

6.3　厚生労働大臣（行政当局）への報告

研究機関の長は、「予測できない」かつ「因果関係が否定できない」かつ「重篤*」な有害事象／不具合について、総長を通して、「（参考書式１）予測できない重篤な有害事象／不具合報告」により FAX で速やかに厚生労働大臣に報告する。

*重篤
死亡、重篤（Grade4)、
軽症/中等症/重症（Grade1/2/3）で治療のための入院又は入院期間の延長が必要となるもの

6.4　効果安全性評価委員会への対応

効果安全性評価委員会を設置した研究の場合、研究責任医師（研究代表者）は、以下について効果安全性評価委員会に審査を依頼する。

1. 情報の評価
2. 計画変更の要否
3. 研究継続の可否
4. その他（新規登録の中断、説明同意文書の改訂、他の被験者への再同意　等）

6.5　情報の公開

(1) 研究機関の長は、予測できない重篤な有害事象への対応と結果を公表する。

(2) 研究機関の長は、現在実施しているまたは過去に実施された人を対象とする医学系研究について、統合指針への重大な不適合を知った場合、速やかに倫理委員会の意見を聴き、必要な対応をした上で、その対応の状況・結果を厚生労働大臣等に報告し、公表する。

7　調査項目・方法

7.1　主要評価項目

評価は、研究前、研究 4 週間後、研究 12 週間後に行う。

■活動量 (歩数)　現在の歩数計の精度は高く信頼性がある [16]。健常な高齢者を対象として歩数計を用いた疫学的調査によると、1 日あたり 4,000 歩歩くことが精神的健康の維持に、7,000 歩で身体的健康の維持に有効であるとされている [5]。また、厚生労働省は身体活動基準 2013 で高齢者の身体活動の基準値を 10 METs・時/週と定め [17]、これに相当する歩数は 4376 歩/日であるとしている [18] 。

介入研究としては、健常な高齢者 (66.38±8.13 歳, 52 人) を対象に、8 週間の mall walking プログラムを行ったところ、日常の歩数が 5,055±1,374 歩から 5,969±1,543 歩に増加したという研究がある [19]。

研究前、研究 4 週間後、研究 12 週間後に、連続する 7 日間の歩数を 3 軸加速度計で計測する。

■Timed Up and Go　(以下、TUG) は、高さ約 46cm の椅子上での椅子座位から起立、3m 歩行、180 度方向転換、再度 3m 歩行、着座という一連の動作をストップウオッチを用いて計測する [20]。TUG は、検者内信頼性、検者間信頼性が高く、下肢筋力、バランス、歩行能力、日常生活機能との関連が深く、また転倒予測の検査としても用いることができる [21]。

膝関節形成術を施行した患者を対象に、週 2〜3 回の通所リハビリを行ったところ、3 ヶ月後に TUG が 12.4 秒から 8.02 秒に改善した [22] 。本邦でも、地域在住の高齢者および虚弱な高齢者に対しての有用性が確かめられている [23] [24] 。

研究前、研究 4 週間後、研究 12 週間後に、 TUG を計測する。

7.2　副次的評価項目

評価は、研究前、研究 4 週間後、研究 12 週間後に行う。

7.2.1　身体機能評価

■活動量 (MET・時)　活動量計は、1 軸加速度計から 3 軸加速度計が主流になりつつあり、より正確になっている。例えば、山田ら [25] は、1 軸加速度計と 3 軸加速度計を二重標識水法と比較し、3 軸加速度計で低強度のエクササイズを計測する重要性を主張している。一方、Park et al [26] は、Kenz Lifecorder EX、Actimarker、Active Style Pro を比較し、通常速度 (75m/min) であれば正確であるものの、低速度 (55m/min) では、機種によって正確性に差があることがわかっている。高齢者は若者と比較すると、総歩数、総エネルギー消費量などが低く、とくに 2.2METs 以上の身体活動は少ない [27]。しか

し、加速度計は高齢者を対象としておらず、慎重に扱う必要がある [28]。

連続する 7 日間の活動量 (MET・時) を 3 軸加速度計で計測する。

■10 m最大歩行速度　Bowden ら [29] は、歩行速度に基づき <0.4m/s を「室内歩行が自立する可能性あり」(household)、0.4m/s〜0.8m/s を「限られた範囲でなら歩行自立」(limited)、>0.8m/s を「歩行自立」(full) と分類した。サルコペニアの判定でも、0.8m/s が基準となっている [30] [31]。また、後述する握力、今回測定しない開眼片脚立ちよりも、歩行速度が介護依存の予想に適している [32]。なお、本邦では交通信号が歩行速度 1.0m/s で設定されていることが多いため、 1.0m/s が基準として適しているとする提案もある。

■握力　は、全身の筋肉量と相関関係があり、予後を示す指標となっている [33]。握力の測定法でよく用いられるのは、スメドレー式握力計を用いて両手の握力を交互に 2 回ずつ測定し、最も大きい値を採用する。

■Barthel Index　(以下、BI) 1965 年に Mahoney et al. [34] により開発され、後述する FIM が採用されるまでは ADL 評価尺度の中でよく用いられてきた。食事、移乗、整容、トイレ動作、入浴、移動、階段昇降、更衣, 排便自制、排尿自制の 10 項目を、それぞれ自立・部分介助など数段階の自立度で評価する。BI は、検者間の再現性に優れた評価法であるが、症状の改善が捉えにくい弱点を持っている。

■Functional Independence Measure　(以下、FIM) 機能的自立度評価と訳される ADL 評価尺度 [35]。運動項目が 13、認知項目が 5 あり、それぞれ 1〜7 点で評価する。ADL が自立時は 126 点、全介助は 18 点となる。数ある ADL 評価尺度の中でも最も信頼性と妥当性があると言われている。

7.2.2　精神機能評価

■Mini-Mental State Examination　(以下、MMSE) MMSE は、簡便な認知症の重症度を検査する尺度である。見当識、記憶、計算、認知、動作、図形描写などの能力を測定する 11 項目からなっている。11 項目の評価基準は、21〜26 点は軽度の認知症、15〜20 点は軽度から中等度の認知症、10〜14 点は中等度の認知症、10 点未満は重度の認知症である。合計点数が低くなるほど認知障害の程度が重い [36] 。

■Short Form 8　(以下、SF-8) は、広く使用されている健康関連 QOL（HRQOL: Health Related Quality of Life）尺度である SF-36 を簡略化した、(1) 身体機能 (2) 日常役割機能（身体) (3) 体の痛み (4) 全体的健康感 (5) 活力 (6) 社会生活機能 (7) 日常役割機能（精神) (8) 心の健康という、健康の 8 領域を測定することができる尺度 [37]。HRQOL という共通した概念で構成されているので、様々な疾患の健康関連 QOL を測定することができ、疾病の異なる患者間の QOL の比較が可能である。

■EuroQOL 5 Dimension 3 L　(以下、EuroQOL-5D-3L) 患者の QOL を測る方法はたくさんあるが、EuroQOL-5D-3L [38] は非常に簡単であり、また多くの疾患に対応できるために、５つの質問で、QOL が最高の状態 (1.0) から最低の状態 (0.0) まで数値化することができる [39]。医療経済評価の計算によく用いられる。

なお、EuroQOL-5D-3L が各質問項目が 3 段階なのに対し、5 段階にした EuroQOL-5D-5L が開発されている [40]。

7.3　評価の中央判定

評価の中央判定は行わない。

8　登録・割付

8.1　登録

(1) 登録の手順

登録後に、事業所の基本情報から適格基準・除外基準を調査し、条件を満たした対象をランダムにグループ A とグループ B に割付ける。

(2) 登録に際しての注意事項

研究開始後の登録は許容されない。

誤登録・重複登録が判明した場合、速やかに研究事務局に連絡する。

8.2　割付

(1) 割付方法、割付調整因子
ランダム割り付け（ランダム化）の方法は、層別ランダム化を行う。
割り付け調整因子は、年齢、性別、原疾患、要介護度とする。
ランダム割付の詳細な手順は研究者等に開示せず、割付責任者が保管する。
(2) 割付調整因子設定の根拠
年齢、性別、原疾患は、要介護度は、活動量 (歩数) と TUG に影響すると考えられる。

9　予定症例数、設定根拠

9.1　予定症例数

予定される症例数は、グループ A が 60 名、グループ B が 60 名であり、合計 120 名である。

9.2　設定根拠

先行研究を参考にサンプルサイズを算出した。介入後の活動量（歩数）と TUG における有意な改善が出現（$\mu > \mu_0$）し、有意水準 ($\alpha = 0.05$) および効果量 ($\delta = 0.50$)、検定力 ($1 - \beta = 0.80$) として、ランダム化比較試験として、対応あり t 検定で両側検定を実施すると仮定した場合、各グループの対象者数は 50 名であった。約 1 割が脱落するとして、各グループそれぞれ 60 名、合計 120 名を対象とする。

9.3　研究対象者登録見込み

本研究は、サンプルサイズは 100 名となる。約 1 割が脱落するとして、120 名 (グループ A 60 名、グループ B 60 名) の登録を見込んでいる。
小規模多機能型居宅介護事業の登録人数は最大 29 名であり、東京都足立区内には 13 事業所ある。それぞれ約 20 名の利用者がいることから、総勢で 260 名程度いると考えられる。利用者はおおむね要介護 1〜3 である。

10　統計解析

10.1　統計解析の方法

本研究の解析対象は、グループ A (ARP 介入群) と グループ B (対照群) である。全ての測定値は、Shapiro-Wilk 検定で正規性を確認する。その後、群内の介入前後に比較においては正規の有無に従いパラメトリックあるいはノンパラメトリック法の平均比較を実施し、群間の比較においては回帰分析及び共分散分析を実施する。検定は、両側検定とし、$p<0.05$ を有意水準とする。

10.2　中間解析と研究の早期中止

中間解析は行わない。

11　データの管理方法、自己点検の方法

11.1　症例記録（Case Report Form：CRF）の作成

CRF の記載の記入及び訂正は研究実施者が行う。研究者等は各対象者の各研究が終了後、速やかに CRF を作成する。記入方法に関しては、「症例報告書の記載の手引き」に従う。CRF を作成するとき、第三者が判読または理解できること、記入間違いに注意すること、読める字で記録すること、鉛筆書きはしないことに留意する。研究協力者は、原資料が存在しその客観性が保証できる場合は、原資料から CRF に転記することができる。

11.2　CRF の自己点検

(1) 研究者等は、CRF 内容と原資料（生データ等）の整合を確認する。
(2) CRF と原資料に矛盾がある場合、その理由を説明する記録を作成する。
(3) 研究責任医師は、作成された CRF についてその内容を点検し、確認した上で記名・押印又は署名を行う。

11.3　CRF の送付及び保管

研究機関の研究責任者は、作成した CRF を定められた手順にて原本を研究事務局に提出する。提出先は下記とする。
（CRF の提出先）
研究事務局
住所：〒980-8574 宮城県仙台市青葉区星陵町 1-1
東北大学大学院医学系研究科内部障害学分野　医局内
TEL: 022-717-7353

11.4　CRF の修正手順

CRF を訂正する場合、研究機関の研究責任者は CRF の変更又は修正の記録を定められた手順にて提出しその写しを保管する。

12　インフォームド・コンセントを受ける手続

12.1　研究対象者への説明

研究者等は、登録前に研究機関の承認を得た説明文書を研究対象者に渡し、以下の内容を説明する。
（説明文書記載事項）

1. 研究の名称、研究実施について研究機関の長の許可を受けている旨
2. 研究機関、研究責任者
3. 研究の目的、意義
4. 研究の方法、期間
5. 研究対象者として選定された理由
6. 研究対象者に生じる負担並びに予測されるリスク、利益
7. 研究実施・継続に同意した場合も随時これを撤回できる旨
8. 研究実施・継続の不同意・同意撤回により研究対象者等が不利益な取扱いを受けない旨
9. 研究に関する情報公開の方法
10. 研究対象者等の求めに応じ他の研究対象者の個人情報等の保護や研究の独創性の確保に支障がない範囲内で研究計画書、研究の方法に関する資料入手・閲覧方法
11. 個人情報等の取扱い（匿名化する場合はその方法を含む）
12. 試料・情報の保存、廃棄の方法
13. 利益相反に関する状況（研究の資金源、起こり得る利害の衝突、研究者等の関連組織との関わり）
14. 研究対象者等及びその関係者からの相談等への対応
15. 研究対象者等に経済的負担・謝礼がある場合の内容
16. 通常診療を超える医療行為を伴う研究の場合、他の治療方法等に関する事項 該当なし
17. 通常診療を超える医療行為を伴う研究の場合、研究終了後の医療提供に関する対応 該当なし
18. 研究の実施に伴う研究対象者の健康、子孫に受け継がれ得る遺伝的特徴等、重要な知見が得られる可能性がある場合、研究対象者に係る研究結果（偶発的所見を含む）の取扱い 該当なし
19. 侵襲を伴う研究の場合、研究によって生じた健康被害に対する補償の有無、内容
20. 研究対象者から取得された試料・情報について、研究対象者等から同意を受ける時点では特定されない将来の研究のために用いられる可能性または他の研究機関に提供する可能性がある場合の同意を受ける時点において想定される

内容

21. 侵襲（軽微な侵襲を除く）を伴う介入研究の場合、研究対象者の秘密が保全されることを条件に、モニタリング、監査の従事者、倫理委員会が研究対象者に関する試料・情報を閲覧する旨

12.2 同意

研究についての説明を行い、十分に考える時間を与え、研究対象者が試験の内容をよく理解したことを確認した上で、試験への参加について依頼する。研究対象者本人が試験参加に同意した場合、同意文書に研究対象者本人による署名を得る。

同意文書は、1 部を研究機関の研究責任者が保管し、1 部を研究対象者本人に渡す。

13 代諾者等からインフォームド・コンセントを受ける場合の手続

代諾者等からインフォームド・コンセントを受けない。

14 インフォームド・アセントを得る場合の手続

インフォームド・アセントを得る状況は想定していない。

15 個人情報等の取扱い

15.1 個人情報の利用目的

研究の正しい結果を得るために、研究中だけではなく研究終了後も長期間にわたり研究対象者個人を特定して調査を行うこと、取得した情報を適切に管理することを目的として個人情報を利用する。

15.2 利用方法（匿名化の方法）

登録者の同定や照会、登録時に発行される被験者識別コードなどを用いて、特別の場合を除き、登録対象者の氏名、生年月日、住所、電話番号等の個人データを特定できないよう配慮する。特別の場合とは、被験者からの研究同意後の撤回またはデータの末梢依頼、有害な事象が発生した場合を指し、そのときに限り、研究を実施する事業所の管理者が対応表を作成して匿名化を行う。

15.3 安全管理責任体制（個人情報の安全管理措置）

研究機関の研究責任者は、個人情報利用にあたり安全管理対策を講じ情報流出リスクを最小化する。

16 研究対象者に生じる負担、予測されるリスク（起こりうる有害事象を含む）・利益、これらの総合的評価、負担・リスクを最小化する対策

16.1 研究参加に伴って予測される利益と不利益の要約

(1) 予測される利益

グループ A は、意欲が向上し、日常の歩数とバランス能力が改善する可能性がある。また、生活の質 (Quality of Life, QOL) が改善する可能性がある。

グループ B は、直接的な利益はないと予想される。

(2) 予測される危険と不利益

グループ A は、屋外での活動を含むために健康被害が生じる恐れがある。介入日には、体温、血圧を事前に計測し、試験実施施設の医師・看護師が参加の可否を判断する。循環動態の変動に伴うめまい、顔色不良、多量発汗、息切れ、血圧や心拍数の異常などは現れる可能性が考えられる。また、下肢の関節痛や筋肉痛の運動器障害が生じる可能性もある。

グループ B は、直接的な不利益はないと予想される。
有害事象が発生した場合には、医師や看護師が適切に対応し、金銭的補償はしない。

17　試料・情報等の保存・廃棄の方法

17.1　保存

研究責任者は、試料・情報等を以下の通り保存する。

保存者	保存する試料・情報等	保存期間
研究責任者	○人体から取得した試料・情報<br>○研究機関において保存すべき研究に係る文書または原資料<br>○手順書　等<br>○委員名簿	研究終了日から 5 年<br><br>結果公表日から 3 年<br>(いずれか遅い日)

ただし、人体から試料を取得することはない。

17.2　廃棄

研究責任医師、研究機関の研究責任者は、人体から取得した試料・情報等を廃棄する場合、匿名化する。

18　研究の資金源、研究機関の研究に係る利益相反及び個人の収益等、研究者等の研究に関する利益相反に関する状況

本研究は運営費交付金により実施する。研究分担者には有限会社アウトソーに在籍する本学の社会人大学院生が含まれている。本研究では、当該研究分担者が勤務する足立区の小規模多機能型居宅介護じゃすみん扇（有限会社アウトソーが運営）を中心とした足立区内の小規模多機能型居宅介護事業所利用者を対象に実施する。

本研究における利益相反については、世界医師会ヘルシンキ宣言及び人を対象とする医学系研究に関する倫理指針（文部科学省、厚生労働省）において、研究対象者への資金提供、スポンサー、利益相反に関する十分な説明と研究計画書への記載が求められていることを踏まえ、研究計画書及び情報公開文書にも記載するものとする。東北大学の研究者等の利益相反は、東北大学利益相反マネジメント委員会が管理する。

「公園の自主管理」については、じゃすみん扇は足立区による報償費をうけている。報償費を受け取っている利用者は研究対象外とする。

19　知的財産

本研究により得られた結果やデータ、知的財産権は、東北大学に帰属する。具体的な取扱いや配分は協議して決定する。知的財産の帰属先を個人とするか研究機関とするかは、所属研究機関の取り決めに従う。

20　研究に関する情報公開の方法

20.1　研究計画の登録

研究責任医師は、臨床試験登録システム (大学病院医療情報ネットワーク、UMIN-CTR) に研究概要を登録し、研究計画書変更、研究進捗に応じて適宜更新する。

`http://www.umin.ac.jp/ctr/index-j.htm`

20.2　研究結果の登録

研究責任医師は、公開データベース等に研究終了後に研究結果を登録する。ただし、研究対象者等の人権、研究者等の関係者の人権、知的財産保護のため非公開とする事項、個人情報保護の観点から研究に著しく支障が生じるため倫理委員会の意見を受け研究機関の長が許可した事項は非公開とする。

20.3　研究結果の公表

研究責任医師は、研究終了後、研究対象者の個人情報保護に措置を講じた上で、遅滞なく研究結果を医学雑誌等に公表する。結果の最終公表を行った場合、遅滞なく研究機関の長に報告する。

21　研究機関の長への報告内容、方法

研究責任医師は、以下を研究機関の長に**「（様式第 8 号）研究の進捗状況等に関する報告書」**により報告する。

- 研究の進捗状況
- 研究の実施に伴う有害事象の発生状況
- 研究終了／中止、結果の概要

22　研究対象者等、その関係者からの相談等への対応

研究全般に関する問合せ窓口（連絡先）
馬場　美彦

東北大学大学院医学系研究科内部障害学分野
〒980-8574　宮城県仙台市青葉区星陵町 1-1
TEL : 022-717-7353 FAX : 022-717-7355

小規模多機能型居宅介護　じゃすみん扇
〒123-0873 東京都足立区扇 1-31-32
TEL: 03-6807-1278 FAX: 03-6807-1279

プライバシーポリシーに関する問合せ窓口（連絡先）
馬場　美彦

東北大学大学院医学系研究科内部障害学分野
〒980-8574　宮城県仙台市青葉区星陵町 1-1
TEL : 022-717-7353 FAX : 022-717-7355

小規模多機能型居宅介護　じゃすみん扇
〒123-0873 東京都足立区扇 1-31-32
TEL: 03-6807-1278 FAX: 03-6807-1279

23　緊急状況下に研究を実施する場合、要件全ての充足を判断する方法

- 研究対象者に緊急かつ明白な生命の危機が生じている
- 通常診療では十分な効果が期待できず、介入研究により研究対象者の生命の危機回避の可能性が十分ある
- 研究実施に伴い研究対象者に生じる負担・リスクが必要最小限度である
- 代諾者となるべき者と直ちに連絡を取れない

24　研究対象者等に経済的負担または謝礼がある場合、その旨、その内容

「公園の自主管理」については、じゃすみん扇は足立区による報償費をうけている。報償費を受け取っている利用者は研究対象外とする。

25　侵襲を伴う研究の場合、研究によって生じた健康被害に対する補償の有無、内容

研究の実施に起因して研究対象者に健康被害が発生した場合、研究機関は治療その他必要な措置を講じる。金銭的な補償はない。

医療費の自己負担分については研究対象者の負担とする。

26　業務内容、委託先の監督方法

研究の業務を委託することはない。

27　試料・情報が同意を受ける時点では特定されない将来の研究のために用いられる可能性／他の研究機関に提供する可能性がある場合、その旨と同意を受ける時点において想定される内容

本研究で得られたデータを二次利用することはない。

28　モニタリングの実施体制

研究責任医師は、試験が安全にかつ研究計画書に従って実施されているか、データが正確に収集されているかの確認を目的として、データ管理者、モニタリング従事者にモニタリングを依頼する。

研究責任医師は、モニタリング計画書を作成し、データ管理者、モニタリング従事者は、モニタリング計画書に基づきモニタリングを実施する。

データ管理者、モニタリング従事者は、モニタリングレポートを研究責任医師に提出する。

① モニタリングは、データ管理者による CRF の記入データに基づくモニタリング従事者による施設訪問にて原資料との照合を含めて行う実地モニタリングを指す。

② モニタリングの目的は、問題点をフィードバックして試験の科学性倫理性を高めることであり、試験や施設の問題点の摘発を意図したものではないため、研究責任医師はモニタリングレポートを検討し、指摘された問題点を研究機関の研究責任者、研究者等と情報共有し、改善に努める。データ管理者、モニタリング従事者は、主に以下の有無についてモニタリングを実施する。

〇違反（violation）
研究計画書に従って行われなかった事象で、以下の複数に該当する事象を「違反」とする。
試験のエンドポイントの評価に実質的な影響を及ぼす
故意または系統的
危険または逸脱の程度が著しい

〇逸脱（deviation）
研究計画書に従って行われなかった事象を「逸脱」とする。
ただし、研究計画書に従うことにより医学的に危険と判断され、研究者の医学的判断に従って治療変更を行った場合、「臨床的に妥当な逸脱」として取扱う。

〇許容範囲の逸脱（acceptable deviation）
研究計画書の許容範囲内の逸脱を「許容範囲の逸脱」とする。

③ 臨床的に妥当な逸脱が多発する場合、研究計画書改訂を検討する。

④ 逸脱の許容範囲設定は、許容範囲内の系統的偏りが試験結果に影響を及ぼす可能性もあるため、望ましいとは限らない。試験の性質、研究者等が研究にどの程度精通するか等を加味し判断する。

⑤ 論文公表の際、原則として違反内容を記載する。特定の逸脱が多い場合は逸脱内容を記載することが望ましい。

29　研究計画書の変更

研究計画書を変更する場合、研究責任医師、研究機関の研究責任者は、倫理委員会の審査を経て研究機関の長の承認を得る。研究計画書内容の変更を、改正・改訂の 2 種類に分けて取扱う。その他、研究計画書の変更に該当しない補足説明の追加をメモランダムとして区別する。

(1) 改正（Amendment）
研究対象者の危険を増大させる可能性のある、または主要評価項目に影響を及ぼす研究計画書の変更。各研究機関の承認を要する。以下の場合が該当する。

- 被験者に対する負担を増大させる変更（採血、検査等の侵襲の増加）
- 重篤な副作用の発現による除外基準等の変更
- 有効性・安全性の評価方法の変更
- 症例数の変更

(2) 改訂（Revision）
研究対象者の危険を増大させる可能性がなく、かつ主要評価項目に影響を及ぼさない研究計画書の変更。各研究機関の承認を要する。以下の場合が該当する。

- 被験者に対する負担を増大させない変更 (検査時期の変更)
- 研究期間の変更
- 研究者の変更

(3) メモランダム／覚え書き（Memorandum）
研究計画書内容の変更ではなく、文面の解釈上のバラツキを減らす、特に注意を喚起する等の目的で、研究責任医師から研究関係者に配布する研究計画書の補足説明。

30　研究の実施体制

30.1　研究機関の名称、研究責任医師の氏名

研究責任者

教授　上月　正博
東北大学大学院医学系研究科内部障害学分野
〒980-8574　仙台市青葉区星陵町 1-1
TEL　022-717-7351　　FAX 022-717-7355
E-mail　kohzuki @ med.tohoku.ac.jp

30.2　共同研究機関

(1) 共同研究機関：なし

30.3　研究事務局、共同研究機関、研究責任者の役割・責任

(1) 研究事務局
東北大学大学院医学系研究科内部障害学分野
〒980-8574　宮城県仙台市青葉区星陵町 1-1

TEL : 022-717-7353 FAX : 022-717-7355
E-mail: babayoshihiko@mac.com

(2) 研究実施責任者
東北大学大学院医学系研究科内部障害学分野
教授　上月　正博

(3) 研究分担者 (学内)
東北大学大学院医学系研究科内部障害学分野
助手　井添　洋輔
医員　大山　千佳
助教　田澤　泰
大学院生　馬場　美彦

30.4　効果安全性評価委員会

1 東北福祉大学　健康科学部・リハビリテーション学科
准教授　五百川　和明
2 東北福祉大学　健康科学部・医療経営管理学科
准教授　河村　孝幸

30.5　統計解析、データセンター

(1) 統計解析責任者
東北大学大学院医学系研究科内部障害学分野
大学院生　馬場　美彦
〒980-8574　仙台市青葉区星陵町 1-1
TEL　022-717-7353　FAX 022-717-7355

(2) データ管理者
東北大学大学院医学系研究科内部障害学分野
教授　上月　正博

(3) モニタリング従事者
東北大学大学院医学系研究科内部障害学分野
助手　高橋　珠緒

30.6　研究に関する問合せ窓口

(1) 研究対象者（参加者）の登録方法：連絡先　大学院生　馬場　美彦
東北大学大学院医学系研究科内部障害学分野
〒980-8574　仙台市青葉区星陵町 1-1
TEL　022-717-7353　FAX 022-717-7355
E-mail: babayoshihiko@mac.com

(2)　有害事象発生時の対応方法：連絡先　教授　上月　正博
東北大学大学院医学系研究科内部障害学分野
〒980-8574　仙台市青葉区星陵町 1-1
TEL　022-717-7351　FAX 022-717-7355
E-mail: kohzuki@med.tohoku.ac.jp

31　引用文献

参考文献

[1] Hirohisa Imai, Yoshinori Fujii, Yoshiharu Fukuda, Hiroyuki Nakao, and Yuichiro Yahata. Health-related quality of life and beneficiaries of long-term care insurance in japan. *Health Policy*, 85(3):349–355, 2008.

[2] Nanako Tamiya, Haruko Noguchi, Akihiro Nishi, Michael R Reich, Naoki Ikegami, Hideki Hashimoto, Kenji Shibuya, Ichiro Kawachi, and John Creighton Campbell. Population ageing and wellbeing: lessons from japan's long-term care insurance policy. *The Lancet*, 378(9797):1183–1192, 2011.

[3] 厚生労働省. 高齢者の地域における新たなリハビリテーションの在り方検討会報告書. Technical report, 厚生労働省, 2015.

[4] 金谷さとみ. 地域における生活機能向上のための理学療法. **理学療法ジャーナル**, 38(7):529–535, 2004.

[5] Yukitoshi Aoyagi and Roy J Shephard. Habitual physical activity and health in the elderly: the nakanojo study. *Geriatrics & Gerontology International*, 10(s1):S236–S243, 2010.

[6] Satoru Kanemori, Yuko Kai, Jun Ida, Katsunori Kondo, Ichiro Kawachi, Hiroshi Hirai, Kokoro Shirai, Yoshiki Ishikawa, and Kayo Suzuki. Social participation and the prevention of functional disability in older japanese: the jages cohort study. *PLoS ONE*, 9(6):e99638, 2014.

[7] 入江多津子. リハビリテーションとケアマネジメント. **総合リハビリテーション**, 38(6):519–525, 2010.

[8] 山永裕明, 野尻晋一, 中西亮二, 桂賢一, 渡邊進, and 米満弘之. 介護保険下の脳卒中維持期リハビリテーション. **リハビリテーション医学**, 42(1):58–71, 2005.

[9] Takako Itsukaichi, Yoshimi Sukukamo, and Shin-Ichi Izumi. Factors influeincing the planning of home-based rehabilitation services by care managers. *Japanese Journal of Comprehensive Rehabilitation Science*, 4:39–46, 2013.

[10] 須貝佑一 and 小林奈美. 施設における痴呆高齢者の転倒・転落事故の発生状況と対策. **看護学雑誌**, 68(1):10–18, 2004.

[11] 川越雅弘, 備酒伸彦, and 森山美知子. 要介護高齢者に対する退院支援プロセスへのリハビリテーション職種の関与状況: 急性期病床，回復期リハビリテーション病床，療養病床間の比較. **理学療法科学**, 26(3):387–392, 2011.

[12] 島田裕之, 内山靖, and 加倉井周一. 21 か月間の縦断研究による虚弱高齢者の転倒頻度と身体機能変化との関係. **総合リハビリテーション**, 30(10):935–941, 2002.

[13] 三浦研, 川越雅弘, and 孔相權. 要介護度および施設種別からみた歩行・移動に関する実態とその環境整備に関する基礎的研究–同一地域におけるアンケート調査から. **生活科学研究誌**, 6, 2007.

[14] Mary E Tinetti and Chandrika Kumar. The patient who falls:" "it's always a trade-off". *JAMA*, 303(3):258–266, 2010.

[15] Anne Forster, Ruth Lambley, Jo Hardy, John Young, Jane Smith, John Green, and Eileen Burns. Rehabilitation for older people in long-term care. *The Cochrane Library*, 2009.

[16] 高戸仁郎, 植木章三, 島貫秀樹, and 芳賀博. 携帯型歩数計を用いた高齢者の歩行能力評価法の開発. **保健福祉学研究**, 2:22–30, 2004.

[17] 厚生労働省. **健康づくりのための身体活動基準２０１３**. 厚生労働省, 2013.

[18] 大須賀洋祐, 薮下典子, 清野諭, 大久保善郎, 鄭松伊, 根本みゆき, フィゲロアラファエル, and 田中喜代次. 高齢者の身体活動基準に相当する 1 日あたりの歩数. **体力科学**, 64(2):243–250, 2015.

[19] S Nicole Culos-Reed, Lynette Stephenson, Patricia K Doyle-Baker, and James A Dickinson. Mall walking as a physical activity option: results of a pilot project. *Canadian Journal on Aging*, 27(01):81–87, 2008.

[20] Diane Podsiadlo and Sandra Richardson. The timed "up & go": a test of basic functional mobility for frail elderly persons. *Journal of the American Geriatrics Society*, 39(2):142–148, 1991.

[21] Anne Shumway-Cook, Sandy Brauer, and Marjorie Woollacott. Predicting the probability for falls in community-dwelling older adults using the timed up & go test. *Physical Therapy*, 80(9):896–903, 2000.

[22] Stephanie C Petterson, Ryan L Mizner, Jennifer E Stevens, LEO Raisis, Alex Bodenstab, William Newcomb, and Lynn Snyder-Mackler. Improved function from progressive strengthening interventions after total knee arthroplasty: a randomized clinical trial with an imbedded prospective cohort. *Arthritis Care & Research*, 61(2):174–183,

2009.

[23] 島田裕之, 古名丈人, 大渕修一, 杉浦美穂, 吉田英世, 金憲経, 吉田祐子, 西澤哲, and 鈴木隆雄. 高齢者を対象とした地域保健活動における timed up & go test の有用性. **理学療法学**, 33(3):105–111, 2006.

[24] 橋立博幸 and 内山靖. 虚弱高齢者における timed “up and go” test の臨床的意義. **理学療法学**, 32(2):59–65, 2005.

[25] Yosuke Yamada, Keiichi Yokoyama, Risa Noriyasu, Tomoaki Osaki, Tetsuji Adachi, Aya Itoi, Yoshihiko Naito, Taketoshi Morimoto, Misaka Kimura, and Shingo Oda. Light-intensity activities are important for estimating physical activity energy expenditure using uniaxial and triaxial accelerometers. *European Journal of Applied Physiology*, 105(1):141–152, 2009.

[26] Jonghoon Park, Kazuko Ishikawa-Takata, Shigeho Tanaka, Yuko Mekata, and Izumi Tabata. Effects of walking speed and step frequency on estimation of physical activity using accelerometers. *Journal of Physiological Anthropology*, 30(3):119–127, 2011.

[27] 樋口博之, 綾部誠也, 進藤宗洋　and 吉武裕, and 田中宏暁. 加速度センサーを内蔵した歩数計による若年者と高齢者の日常活動量の比較. **体力科学**, 52:111–118, 2003.

[28] Jennifer A Schrack, Rachel Cooper, Annemarie Koster, Eric J Shiroma, Joanne M Murabito, W Jack Rejeski, Luigi Ferrucci, and Tamara B Harris. Assessing daily physical activity in older adults: Unraveling the complexity of monitors, measures, and methods. *The Journals of Gerontology Series A*, page glw026, 2016.

[29] Mark G. Bowden, Chitralakshmi K. Balasubramanian, Andrea L. Behrman, and Steven A. Kautz. Validation of a speed-based classification system using quantitative measures of walking performance poststroke. *Neurorehabilitation and Neural Repair*, 2008.

[30] Liang-Kung Chen, Li-Kuo Liu, Jean Woo, Prasert Assantachai, Tung-Wai Auyeung, Kamaruzzaman Shahrul Bahyah, Ming-Yueh Chou, Liang-Yu Chen, Pi-Shan Hsu, Orapitchaya Krairit, et al. Sarcopenia in asia: consensus report of the asian working group for sarcopenia. *Journal of the American Medical Directors Association*, 15(2):95–101, 2014.

[31] Alfonso J. Cruz-Jentoft, Jean Pierre Baeyens, Jürgen M. Bauer, Yves Boirie, Tommy Cederholm, Francesco Landi, Finbarr C. Martin, Jean-Pierre Michel, Yves Rolland, Stéphane M. Schneider, Eva Topinková, Maurits Vandewoude, and Mauro Zamboni. Sarcopenia: European consensus on definition and diagnosis: Report of the european working group on sarcopenia in older people. *Age and Ageing*, page afq034, 2010.

[32] S. Shinkai, S. Watanabe, S. Kumagai, Y. Fujiwara, H. Amano, H. Yoshida, and H. Shibata. Walking speed as a good predictor for the onset of functional dependence in a japanese rural community population. *Age and Ageing*, 29(5):441–446, 2000.

[33] Y. Fujita, Y. Nakamura, J. Hiraoka, K. Kobayashi, K. Sakata, M. Nagai, and H. Yanagawa. (1995). physical-strength tests and mortality among visitors to health-promotion centers in japan. *Journal of Clinical Epidemiology*, 48(11):1349–1359, 1995.

[34] F. I. Mahoney. Functional evaluation: the barthel index. *Maryland State Medical Journal*, 14:61–65, 1965.

[35] RA Keith, CV Granger, BB Hamilton, and FS Sherwin. The functional independence measure. *Adv Clin Rehabil*, 1:6–18, 1987.

[36] Marshal F. Folstein, Susan E. Folstein, and Paul R. McHugh. “mini-mental state”: a practical method for grading the cognitive state of patients for the clinician. *Journal of Psychiatric Research*, 12(3):189–198, 1975.

[37] S. Fukuhara and Y. Suzukamo. *Manual of the SF-8 Japanese version*. Institute for Health Outcomes & Process Evaluation Research, Kyoto, 2004.

[38] 西村周三, 土屋有紀, 久繁哲徳, 池上直己, and 池田俊也. 日本語版 euroqol の開発. **医療と社会**, 8(1):109–123, 1998.

[39] Aki Tsuchiya, Shunya Ikeda, Naoki Ikegami, Shuzo Nishimura, Ikuro Sakai, Takashi Fukuda, Chisato Hamashima, Akinori Hisashige, and Makoto Tamura. Estimating an eq-5d population value set: the case of japan. *Health Economics*, 11(4):341–353, 2002.

[40] 池田俊也, 白岩健, 五十嵐中, 能登真一, 福田敬, 齋藤信也, and 下妻晃二郎. 日本語版 eq-5d-5l におけるスコアリング法の開発. **保健医療科学**, 64(1):47–55, 2015.

32 Appendix

被験者登録番号	
割付群	□ グループ A　　□ グループ B
施設名	
報告書作成日	（西暦）　　年　月　日
報告書作成者名	印
責任医師名	印

作成日　第　版　　月　月　日

〔記入上の注意〕

1. 記入は、黒色のボールペンで行う。
2. 訂正は、訂正前の記入内容が分かるように二重線を引き、訂正日（年月日）と訂正理由の記入と試験責任医師又は試験分担医師の捺印を行う。
3. 空欄には「記入漏れ」と区別するために、その理由（実施せず・未実施）または斜線を入れる。

【主要評価項目】

歩数 (一日の歩数を記録)　　年　月

日	歩数/活動量	日	歩数/活動量	日	歩数/活動量
1	/	11	/	21	/
2	/	12	/	22	/
3	/	13	/	23	/
4	/	14	/	24	/
5	/	15	/	25	/
6	/	16	/	26	/
7	/	17	/	27	/
8	/	18	/	28	/
9	/	19	/	29	/
10	/	20	/	30	/
	/		/	31	/

TUG	秒	月　日

【副次的評価項目】

握力	kg	MMSE			
10m 最大歩行	秒	BI		EuroQOL	
	歩	FIM		SF-8	

【臨床所見】

脈拍	bpm
自覚症状	□なし　□あり (下記に詳細を記入)

被験者名：　　　　　　　　　　様	検査者名：	日付

Barthel Index (研究者記録用)

食事	自立、自助具などの装着可、標準時間内に食べ終える<br>部分介助 (たとえば、おかずを切って細かくしてもらう)<br>全介助	10 点<br>5 点<br>0 点
車椅子からベッドへの移乗	自立、ブレーキ、フットレストの操作も含める<br>軽度の部分介助または監視を要する<br>座ることは可能であるがほぼ全介助<br>全介助または不可能	15 点<br>10 点<br>5 点<br>0 点
整容動作	洗面、整髪、歯磨き、髭剃りなどが自立<br>部分介助または不可能	5 点<br>0 点
トイレ動作	自立 (衣服の操作、後始末を含む)<br>部分介助、体を支える、衣服、後始末に介助を要する<br>全介助または不可能	10 点<br>5 点<br>0 点
入浴	自立<br>全介助または不可能	5 点<br>0 点
歩行・車椅子の移動	45m 以上の歩行が可能 (自助具の使用可)<br>45m 以上の介助歩行ができる (自助具可)<br>歩行不能の場合、車椅子にて 45m 以上の操作可能<br>上記以外	15 点<br>10 点<br>5 点<br>0 点
階段昇降	自立、手すり等の使用の有無は問わない<br>介助あるいは監視を要する<br>不能	10 点<br>5 点<br>0 点
更衣動作	自立、靴・ジッパー・装具の着脱を含む<br>上記以外	10 点<br>0 点
排便コントロール	失禁なし、浣腸・座薬の取り扱いも可能<br>時に失禁あり、浣腸・座薬の取り扱いに介助を要する者も含む<br>上記以外	10 点<br>5 点<br>0 点
排尿コントロール	失禁なし、収尿器の取り扱いも可能<br>時に失禁あり、収尿器の取り扱いに介助を要する者も含む。<br>上記以外。	10 点<br>5 点<br>0 点
合計		点

被験者名： 様	検査者名：	日付

FIM（研究者記録用）

運動項目		
食事	口に運ぶ動作、咀嚼、嚥下を含めた食事動作	
整容	口腔ケア、整髪、手洗い、洗顔、髭剃り・化粧など	
清拭	風呂、シャワーなどで首から下（背中以外）を洗う	
更衣・上半身	腰より上の更衣および義肢装具の装着	
更衣・下半身	腰より下の更衣および義肢装具の装着	
トイレ動作	衣服の着脱、排泄後の清潔、生理用具の使用	
排尿管理	排尿の管理、器具や薬剤の使用を含む、失敗の頻度	
排便管理	排便の管理、器具や薬剤の使用を含む、失敗の頻度	
ベッド・椅子・車椅子	それぞれの間の移乗、起立動作を含む	
トイレ	便器へ（から）の移乗	
浴槽・シャワー	浴槽、シャワー室へ（から）の移乗	
歩行 (車椅子)	屋内での歩行 (車椅子移動)	
階段	12 − 14 段の階段昇降	
認知項目		
理解	聴覚または視覚によるコミュニケーションの理解	
表出	言語的または非言語的表現	
社会的交流	他患、スタッフなどとの交流、社会的状況への順応	
問題解決	日常生活上での問題解決、適切な決断能力	
記憶	日常生活に必要な情報の記憶	
合計		点

運動項目
7: 完全自立 (時間、安全性含め)
6: 修正自立 (時間がかかる、補助具使用、安全性配慮)
5: 監視・準備
4: 最小介助 (患者自身で 75% 以上)
3: 中等度介助 (患者自身で 50% 以上)
2: 最大介助 (患者自身で 25% 以上)
1: 全介助 (患者自身で 25% 未満)

認知項目
7: 完全自立 (時間、安全性含め)
6: 修正自立 (時間がかかる、補助具使用、安全性配慮)
5: 監視・準備 (患者自身で 90% 以上)
4: 最小介助 (患者自身で 75% 以上)
3: 中等度介助 (患者自身で 50% 以上)
2: 最大介助 (患者自身で 25% 以上)
1: 全介助 (患者自身で 25% 未満)

Mini-Mental State Examination（MMSE）

得点：30 点満点

検査日：２００　年　月　日　曜日　施設名：

被験者：　　　　男・女　生年月日：明・大・昭　年　月　日　歳

プロフィールは事前または事後に記入します。　検査者：

	質問と注意点	回　答	得　点
1（５点）<br>時間の<br>見当識	**「今日は何日ですか」**<br>＊最初の質問で、被験者の回答に複数の項目が含まれていてもよい。その場合、該当する項目の質問は省く。	日	０　１
	**「今年は何年ですか」**	年	０　１
	**「今の季節は何ですか」**		０　１
	**「今日は何曜日ですか」**	曜日	０　１
	**「今月は何月ですか」**	月	０　１
2（５点）<br>場所の<br>見当識	**「ここは都道府県でいうと何ですか」**		０　１
	**「ここは何市（＊町・村・区など）ですか」**		０　１
	**「ここはどこですか」**<br>（＊回答が地名の場合、この施設の名前は何ですか、と質問をかえる。正答は建物名のみ）		０　１
	**「ここは何階ですか」**	階	０　１
	**「ここは何地方ですか」**		０　１
3（３点）<br>即時想起	**「今から私がいう言葉を覚えてくり返し言ってください。『さくら、ねこ、電車』はい、どうぞ」**<br>＊テスターは３つの言葉を１秒に１つずつ言う。その後、被験者にくり返させ、この時点でいくつ言えたかで得点を与える。<br>＊正答１つにつき１点。合計３点満点。<br>**「今の言葉は、後で聞くので覚えておいてください」**<br>＊この３つの言葉は、質問５で再び復唱させるので３つ全部答えられなかった被験者については、全部答えられるようになるまでくり返す（ただし６回まで）。		０　１<br>２　３
4（５点）<br>計算	**「１００から順番に７をくり返しひいてください」**<br>＊５回くり返し７を引かせ、正答１つにつき１点。合計５点満点。<br>正答例：９３　８６　７９　７２　６５<br>＊答えが止まってしまった場合は「それから」と促す。		０ １ ２<br>３ ４ ５
5（３点）<br>遅延再生	**「さっき私が言った３つの言葉は何でしたか」**<br>＊質問３で提示した言葉を再度復唱させる。		０ １ ２ ３
6（２点）<br>物品呼称	時計（又は鍵）を見せながら**「これは何ですか？」**<br>鉛筆を見せながら**「これは何ですか？」**<br>＊正答１つにつき１点。合計２点満点。		０ １ ２
7（１点）<br>文の復唱	**「今から私がいう文を覚えてくり返し言ってください。『みんなで力を合わせて綱を引きます』」**<br>＊口頭でゆっくり、はっきりと言い、くり返させる。１回で正確に答えられた場合１点を与える。		０　１
8（３点）<br>口頭指示	＊紙を机に置いた状態で教示を始める。<br>**「今から私がいう通りにしてください。右手にこの紙を持ってください。それを半分に折りたたんでください。そして私にください」**<br>＊各段階毎に正しく作業した場合に１点ずつ与える。合計３点満点。		０ １ ２ ３
9（１点）<br>書字指示	**「この文を読んで、この通りにしてください」**<br>＊被験者は音読でも黙読でもかまわない。実際に目を閉じれば１点を与える。	裏面に質問有	０　１
10（１点）<br>自発書字	**「この部分に何か文章を書いてください。どんな文章でもかまいません」**<br>＊テスターが例文を与えてはならない。意味のある文章ならば正答とする。（＊名詞のみは誤答、状態などを示す四字熟語は正答）	裏面に質問有	０　１
11（１点）<br>図形模写	**「この図形を正確にそのまま書き写してください」**<br>＊模写は角が１０個あり、２つの五角形が交差していることが正答の条件。手指のふるえなどはかまわない。	裏面に質問有	０　１

９.「この文を読んで、この通りにしてください」

「目（め）を閉（と）じてください」

１０.「この部分に何か文章を書いてください。どんな文章でもかまいません」

１１.「この図形を正確にそのまま書き写してください」

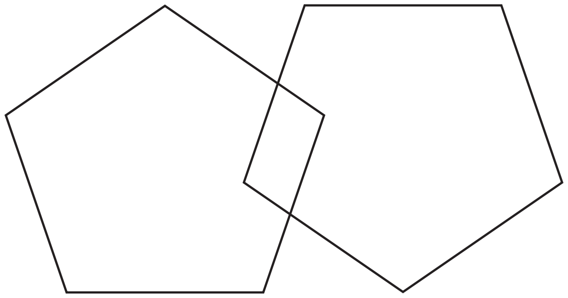

あなたの健康について

このアンケートはあなたがご自分の健康をどのように考えているかをおうかがいするものです。あなたが毎日をどのように感じ、日常の活動をどのくらい自由にできるかを知るうえで参考になります。お手数をおかけしますが、何卒ご協力のほど宜しくお願い申し上げます。

以下のそれぞれの質問について、一番よくあてはまるものに印（☑）をつけてください。

1. 全体的にみて、過去１ヵ月間のあなたの健康状態はいかがでしたか。

最高に良い	とても良い	良い	あまり良くない	良くない	ぜんぜん良くない
▼	▼	▼	▼	▼	▼
☐ 1	☐ 2	☐ 3	☐ 4	☐ 5	☐ 6

2. 過去１ヵ月間に、体を使う日常活動（歩いたり階段を昇ったりなど）をすることが身体的な理由でどのくらい妨（さまた）げられましたか。

ぜんぜん、妨（さまた）げられなかった	わずかに妨（さまた）げられた	少し妨（さまた）げられた	かなり、妨（さまた）げられた	体を使う日常活動ができなかった
▼	▼	▼	▼	▼
☐ 1	☐ 2	☐ 3	☐ 4	☐ 5

3. 過去１ヵ月間に、いつもの仕事（家事も含みます）をすることが、身体的な理由でどのくらい妨（さまた）げられましたか。

ぜんぜん、妨（さまた）げられなかった	わずかに妨（さまた）げられた	少し妨（さまた）げられた	かなり、妨（さまた）げられた	いつもの仕事ができなかった
▼	▼	▼	▼	▼
☐ 1	☐ 2	☐ 3	☐ 4	☐ 5

サンプル

SF-8™ Health Survey (Japanese version) Copyright © 1999, 2001, 2003 by QualityMetric, Inc. and Shunichi Fukuhara All rights reserved.

4.　過去１ヵ月間に、体の痛みはどのくらいありましたか。

ぜんぜんなかった	かすかな痛み	軽い痛み	中くらいの痛み	強い痛み	非常に激しい痛み
☐ 1	☐ 2	☐ 3	☐ 4	☐ 5	☐ 6

5.　過去１ヵ月間、どのくらい元気でしたか。

非常に元気だった	かなり元気だった	少し元気だった	わずかに元気だった	ぜんぜん元気でなかった
☐ 1	☐ 2	☐ 3	☐ 4	☐ 5

6.　過去１ヵ月間に、家族や友人とのふだんのつきあいが、身体的あるいは心理的な理由で、どのくらい妨(さまた)げられましたか。

ぜんぜん、妨(さまた)げられれなかった	わずかに、妨(さまた)げられた	少し、妨(さまた)げられた	かなり、妨(さまた)げられた	つきあいができなかった
☐ 1	☐ 2	☐ 3	☐ 4	☐ 5

7.　過去１ヵ月間に、心理的な問題（不安を感じたり、気分が落ち込んだり、イライラしたり）に、どのくらい悩まされましたか。

ぜんぜん悩まされなかった	わずかに悩まされた	少し悩まされた	かなり悩まされた	非常に悩まされた
☐ 1	☐ 2	☐ 3	☐ 4	☐ 5

8.　過去１ヵ月間に、日常行う活動（仕事、学校、家事などのふだんの行動）が、心理的な理由で、どのくらい妨(さまた)げられましたか。

ぜんぜん、妨(さまた)げられなかった	わずかに、妨(さまた)げられた	少し、妨(さまた)げられた	かなり、妨(さまた)げられた	日常行う活動ができなかった
☐ 1	☐ 2	☐ 3	☐ 4	☐ 5

ご協力、ありがとうございました。

SF-8™ Health Survey (Japanese version) Copyright © 1999, 2001, 2003 by QualityMetric, Inc. and Shunichi Fukuhara　All rights reserved.

被験者名：　　　　　　　　　　様	検査者名：	日付

EuroQOL (EQ-5D-3L) (研究者記録用)

移動の程度	私は歩き回るのに問題はない	1
	私は歩き回るのに問題がある	2
	私はベッド（床）に寝たきりである	3
身の回りの管理	私は身の回りの管理に問題はない	1
	私は洗面や着替えを自分でするのにいくらか問題がある	2
	私は洗面や着替えを自分でできない	3
ふだんの活動	私はふだんの活動を行うのに問題はない	1
（例：仕事、勉強、家族・余暇活動）	私はふだんの活動を行うのにいくらか問題がある	2
	私はふだんの活動を行うことができない	3
痛み/不快	私は痛みや不快感はない	1
	私は中程度の痛みや不快感がある	2
	私はひどい痛みや不快感がある	3
不安/ふさぎ込み	私は不安でもふさぎ込んでもいない	1
	私は中程度に不安あるいはふさぎ込んでいる	2
	私はひどく不安あるいはふさぎ込んでいる	3
結果<br>(結果は 11223 のように記す。)		

11111 1.000
11112 0.786
11113 0.736
11121 0.768
11122 0.705
11123 0.656
11131 0.654
11132 0.592
11133 0.542
11211 0.804
11212 0.742
11213 0.692
11221 0.724
11222 0.661
11223 0.612
11231 0.610
11232 0.548
11233 0.498
11311 0.715
11312 0.652
11313 0.603
11321 0.635
11322 0.572
11323 0.522
11331 0.521
11332 0.458
11333 0.409
12111 0.795
12112 0.732
12113 0.682
12121 0.714
12122 0.652
12123 0.602
12131 0.601
12132 0.538
12133 0.488
12211 0.751
12212 0.688
12213 0.638
12221 0.670
12222 0.608
12223 0.558
12231 0.557
12232 0.494
12233 0.444
12311 0.661
12312 0.599
12313 0.549
12321 0.581
12322 0.518
12323 0.469
12331 0.467
12332 0.405
12333 0.355
13111 0.747
13112 0.684
13113 0.634
13121 0.666
13122 0.604
13123 0.554
13131 0.553
13132 0.490
13133 0.440
13211 0.703
13212 0.640
13213 0.590
13221 0.622
13222 0.560
13223 0.510
13231 0.509
13232 0.446
13233 0.396
13311 0.614
13312 0.551
13313 0.501
13321 0.533
13322 0.470
13323 0.421
13331 0.419
13332 0.357
13333 0.307
21111 0.774
21112 0.711
21113 0.661
21121 0.693
21122 0.631
21123 0.581
21131 0.580
21132 0.517
21133 0.467
21211 0.730
21212 0.667
21213 0.617
21221 0.649
21222 0.587
21223 0.537
21231 0.536
21232 0.473
21233 0.423
21311 0.640
21312 0.578
21313 0.528
21321 0.560
21322 0.497
21323 0.448
21331 0.446
21332 0.384
21333 0.334
22111 0.720
22112 0.657
22113 0.608
22121 0.640
22122 0.577
22123 0.527
22131 0.526
22132 0.463
22133 0.414
22211 0.676
22212 0.613
22213 0.564
22221 0.596
22222 0.533
22223 0.483
22231 0.482
22232 0.419
22233 0.370
22311 0.587
22312 0.524
22313 0.474
22321 0.506
22322 0.444
22323 0.394
22331 0.393
22332 0.330
22333 0.280
23111 0.672
23112 0.609
23113 0.560
23121 0.592
23122 0.529
23123 0.479
23131 0.478
23132 0.415
23133 0.366
23211 0.628
23212 0.565
23213 0.516
23221 0.548
23222 0.485
23223 0.435
23231 0.434
23232 0.371
23233 0.322
23311 0.539
23312 0.476
23313 0.426
23321 0.459
23322 0.396
23323 0.346
23331 0.345
23332 0.282
23333 0.232
31111 0.430
31112 0.367
31113 0.318
31121 0.350
31122 0.287
31123 0.237
31131 0.236
31132 0.173
31133 0.124
31211 0.386
31212 0.323
31213 0.274
31221 0.306
31222 0.243
31223 0.193
31231 0.192
31232 0.129
31233 0.080
31311 0.297
31312 0.234
31313 0.184
31321 0.216
31322 0.154
31323 0.104
31331 0.103
31332 0.040
31333 -0.010
32111 0.376
32112 0.314
32113 0.264
32121 0.296
32122 0.233
32123 0.184
32131 0.182
32132 0.120
32133 0.070
32211 0.332
32212 0.270
32213 0.220
32221 0.252
32222 0.189
32223 0.140
32231 0.138
32232 0.076
32233 0.026
32311 0.243
32312 0.180
32313 0.131
32321 0.163
32322 0.100
32323 0.050
32331 0.049
32332 -0.014
32333 -0.063
33111 0.328
33112 0.266
33113 0.216
33121 0.248
33122 0.185
33123 0.136
33131 0.134
33132 0.072
33133 0.022
33211 0.284
33212 0.222
33213 0.172
33221 0.204
33222 0.141
33223 0.092
33231 0.090
33232 0.028
33233 -0.022
33311 0.195
33312 0.132
33313 0.083
33321 0.115
33322 0.052
33323 0.002
33331 0.001
33332 -0.062
33333 -0.111
