# Supplementary material for: Effects of Adachi Rehabilitation Programme on older adults under long-term care: A multi-centre controlled trial
Source: PLoS One. 2021 Feb 12;16(2):e0245646. doi: 10.1371/journal.pone.0245646 (PMC7880461; doi:10.1371/journal.pone.0245646)
Supplement: S2 File — (PDF) [file pone.0245646.s006.pdf]

Class II

東北大学

Development and Evaluation of Adachi Rehabilitation Programme on older adults under long term care

Registration: UMIN-CTR UMIN000028317

Principal investigator

Prof. Masahiro Kohzuki
Department of Rehabilitation Science and Internal Medicine
Tohoku University Graduate School of Medicine
Seiryo-cho 1-1, Aoba-ku, Sendai, 980-8574
TEL 022-717-7351 FAX 022-717-7355
E-mail: kohzuki@med.tohoku.ac.jp

Research Bureau

Yoshihiko Baba
Department of Rehabilitation Science and Internal Medicine
Tohoku University Graduate School of Medicine
cho 1-1, Aoba-ku, Sendai, 980-8574
TEL 022-717-7353 FAX 022-717-7355
E-mail: babayoshihiko@mac.com

目次

1 Purpose 6

2 Background and Rationale for the Research Plan 6
2.1 Background 6
2.2 The scientific rationale for the study 7

3 Selection Policy for Research Subjects 8
3.1 Inclusion criteria 8
3.2 Exclusion criteria 8

4 Research method, period 8
4.1 Research design 8
4.2 Details of the treatment and intervention plan 8
4.3 Combination therapy 10
4.4 Post-treatment 10
4.5 Inspection schedule 10
4.6 Research period 10

5 Assessment of Adverse Events 10
5.1 Obtaining Information 11
5.2 Description of Adverse Events 12

6 Response to serious adverse events/failures (including the range of adverse events to be reported to the head of the institution) 12
6.1 Response to Adverse Events/Failures 12
6.2 Report to the head of the institution and the principal investigator (principal investigator) 13
6.3 Report to the Minister of Health, Labour and Welfare (administrative authorities) 13
6.4 Response to the Efficacy and Safety Evaluation Committee 13
6.5 Release of information 14

7 Survey items and methods 14
7.1 Primary outcomes 14
7.2 Secondary outcomes 14
7.3 Interim Judgment of Evaluation 15

8 Registration and Allocation 16
8.1 Registration 16
8.2 Allocation 16

9 Expected number of cases, and its reasons 16
9.1 Expected number of cases 16
9.2 Reasons 16
9.3 Prospective Research Subjects 16

10 Statistics 16
10.1 Statistical methods 16
10.2 Interim analysis and early termination of the study 17

11 Data management and self-assessment methods 17

11.1 Preparation of Case Report Forms (CRFs) . . . 17
11.2 CRF self-inspection . . . 17
11.3 Sending and storing of CRFs . . . 17
11.4 CRF Correction Procedure . . . 17

12 Procedures for obtaining informed consent 17
12.1 Explanation to Research Subjects . . . 17
12.2 Consent . . . 18

13 (Procedures for receiving informed consent from a proxy, etc.) 18

14 Procedures for obtaining an informed assent 19

15 Handling of Personal Information, etc. 19
15.1 Purpose of Use of Personal Information . . . 19
15.2 How to use (anonymization method) . . . 19
15.3 Safety Management System (Safety Management Measures for Personal Information) . . . 19

16 Burden on research subjects, anticipated risks (including possible adverse events) and benefits, a comprehensive assessment of these, and measures to minimise the burden and risk. 19
16.1 Summary of anticipated benefits and disadvantages associated with research participation . . . 19

17 Methods for Storage and Disposal of Samples and Information 20
17.1 Sample preservation . . . 20
17.2 Abandonment . . . 20

18 Conflicts of interest regarding research sources, conflicts of interest relating to research at research institutions, and individual earnings, and conflicts of interest relating to research by researchers and others 20

19 Intellectual Property 20

20 How to publish information about your research 21
20.1 Registering a Research Plan . . . 21
20.2 Registration of Research Results . . . 21
20.3 Publication of Research Results . . . 21

21 Content and method of reporting to the head of the research institution 21

22 Responding to consultations, etc., from research subjects and other related parties 21

23 How to determine the satisfaction of all requirements when conducting research under emergency conditions 22

24 If there are any financial burdens or rewards to be paid to the research subjects or others, please describe them in detail. 22

25 In the case of invasive research, whether and what compensation is available for health hazards caused by the research 22

26 Description of work and supervision of contractors 22

27 The possibility that the sample/information may be used for future research that is not specified at the time the consent is received/probable to be provided to other institutions, if any, and what is anticipated at the time the consent is received 22

28 Monitoring System 22

29 Changes to the Research Proposal 23

30 Research System 24

30.1 The name of the research institution and the name of the principal investigator 24

30.2 Collaborative Research Institute 24

30.3 Roles and responsibilities of research secretariats, collaborating institutions and principal investigators 24

30.4 Efficacy and Safety Evaluation Committee 24

30.5 Statistical Analysis, Data Center 25

30.6 Contact for Research Inquiries 25

31 引用文献 25

32 Appendix 28

Summary

Schema

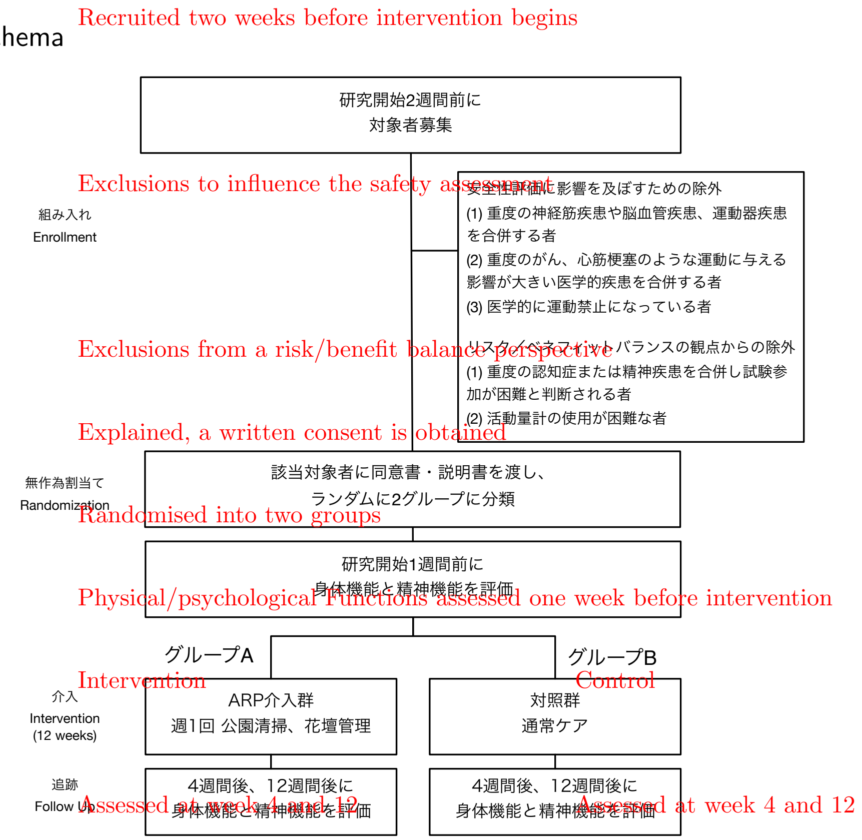

Purpose

For the elderly who use long-term care insurance services, the The Adachi Rehabilitation Programme (ARP) was developed as a community rehabilitation program to promote participation. The effects of ARP on activity and psychosomatic functioning will be tested in a randomized comparative study with usual care.

Participants

Elderly people who need nursing care level 1-3 who are using the small-scale multifunctional at-home care facility in Adachi-ku, Tokyo.

Treatment

The ARP is a community rehabilitation program with weekly participation, one cycle of four weeks.

The first week, once a week, I ride the bus for about 10-20 minutes to go shopping for park cleanup and flower bed maintenance needs. It takes three hours to travel and shop. For the next three weeks, they will spend one hour once a week at a neighborhood park to clean up and manage their flower beds. The park is located in the neighborhood of the nursing home (less than 500 meters away) and is about 1,000m^2 in area with some rolling terrain. Walk to the park (cane and silver car available). Cleanup activities include carrying tongs and garbage bags and moving freely around the park to pick up trash. To maintain the flower beds, digging the soil, planting flowering plants and watering them with a hose. These activities are carried out under Adachi Ward's "Self-management of Parks" and "Self-management of Flowerbeds" systems.

Do this for three cycles (12 weeks). On other days, the participants live as normal.

Group A Perform the above ARP once a week.

Group B While the intervention group is performing the ARP, they are asked to choose from three different activities, mainly seated, to watch TV, colouring books, and folding laundry, as their usual care.

Planned number of cases, Research period

(1) Planned number of cases: a significant improvement in step count and Timed Up and Go emerged, with a significant level ($\alpha = 0.05$) and effect size ($\delta = 0.50$), with Assuming a two-tailed test with a paired t-test as the test power ($1 - \beta = 0.80$), then Each group should have 50 participants, for a total of 100 people.

There were 50 participants in Group A and 50 participants in Group B, with Group A being the ARP intervention group and Group B will be the control group. We estimate that about 10% of the participants will drop out of the group, and we expect 120 participants (60 in Group A and 60 in Group B) to enroll.

(2) Research period: November 2017 (after Ethics Committee approval) - September 2021

Contact

Yoshihiko Baba

Department of Rehabilitation Science and Internal Medicine, Tohoku University Graduate School of Medicine

Seiryo-cho 1-1, Aoba-ku, Sendai, 980-8574

TEL: 022-717-7353 FAX: 022-717-7355

Jasmine Ougi (Small Multifunctional At-home Care)

Ougi 1-31-32, Adachi-ku, Tokyo 123-0873

TEL: 03-6807-1278 FAX: 03-6807-1279

E-mail: babayoshihiko@mac.com

1 Purpose

The Adachi Rehabilitation Program (ARP), which promotes the participation of seniors in need of nursing care (1-3), is The benefits of increased motivation, increased amount of daily physical activity, and improved physical functions such as balance, have been shown to Evaluate by randomized comparison with usual care.

The primary assessments are steps and Timed Up and Go.

2 Background and Rationale for the Research Plan

2.1 Background

In Japan, the long-term care insurance system was launched in 2000 with the aim of supporting the independence of elderly people who need long-term care. The company has been praised for its ability to provide more services at a lower price. [1] [2] However, the quality of home care is still a challenge. [2] In addition, although the transfer of living-phase rehabilitation for the elderly from medical insurance to long-term care insurance is being considered, the Compared to acute and convalescent rehabilitation, it is more difficult to improve physical and mental functions and physical structure. Therefore, the Ministry of Health, Labour and Welfare (MHLW) has been working to improve not only "physical and mental functions and physical structure" but also Recommends "activities" and "participation" such as shopping and outings that utilize residual capacity as rehabilitation. [3]

The in-home service plan (care plan) includes "activities" in daily living such as cooking, "participation" such as a walk in a nearby park, and Opportunities for "participation" such as shopping in shopping malls and using public transportation. Prior to the International Classification of Functioning, Disability and Health (ICF), the Rehabilitation was seen as improving physical and mental functions, such as "going out when I can walk," but The ICF suggests that

‘participation’ may, in turn, improve ‘activity’. [4] This has been confirmed in a large observational study of healthy elderly people. [5] [6] Although it is assumed that promoting “participation” to older people with reduced “activity” would also have an ameliorating effect on “activity” such as increased daily physical activity and balance ability, it is No intervention study has tested the effects of an intervention as small as once a week as in the present study.

Against the above background, it is expected that the programs being implemented in the nursing home will have a rehabilitative effect, but It has not yet been evaluated. Therefore, in this study, the participation program in Adachi Ward’s long-term care facilities is The program is named the Adachi Rehabilitation Programme (ARP). A randomized controlled trial will be conducted to determine the effects of ARPs on ”activity” and ”physical and mental function” in elderly people in need of care.

The ARP is a program for elderly people who need long-term care services. Cleanup and flower bed management in the park, and Using public transportation to go shopping as an intervention. Evaluate the effects of ARP on physical activity, balance ability, cognitive function, quality of life and other physical and mental functions.

The facilities where the research was carried out are the member offices of Adachi Ward Multi-Functional Service Liaison Association, of which the research co-investigator (Yoshihiko Baba) is the sponsor. The Adachi-ku multifunctional service consists of all the small-scale multifunctional at-home care providers (13 service providers) in Adachi-ku.

2.2 The scientific rationale for the study

In order to consider functional training for the elderly provided by the nursing care service as a form of rehabilitation for life, it is necessary to There are several issues that could be addressed/ [7] [8] [9] [10] [11]

First, although rehabilitation needs to be incorporated into care management, the The in-home service plan (care plan) prepared by the long-term care insurance includes Half of those who need nursing care 4 use home rehabilitation, and those who need nursing care 5 have 17% of the total, while those who need nursing care 3 or less have almost no use of home rehabilitation. [7] Home rehabilitation is the most underused service in long-term care insurance [8] and Some point out that care support specialists (care managers) need to be educated, [7] [8] [9]

Secondly, setting up an environment that reduces the risk of falls and other injuries is important for rehabilitation at home and in the community. [12] Although there is research on improving the environment within nursing homes[13], There are no studies that have investigated the environmental arrangements necessary for people in need of care to get out of the house or how going out of the house affects physical and mental functioning of people in need of care. Even in senior care facilities, falls are common. [10] Rehabilitation at home or in the community comes with the risk of falls. [14]

Furthermore, according to a systematic review of rehabilitation in long-term care, 30 of the 49 studies were in the United States and Also, the subject must be a resident of the facility. [15] This suggests that the environment is different from that of care at home in Japan. Research on long-term care insurance service users in Japan has been limited to stroke patients, for example, and It is argued that although it has been shown to improve the activities of daily living (ADL), there is insufficient scientific evidence to support this claim. [8] At the time of discharge from the hospital, care conferences are held between rehabilitation professionals and caregivers, but Some reports indicate that there is little research on post-discharge rehab and its outcomes.[11]

Based on the above, we believe that the development of a program that can be located in a care plan and that can be performed in long-term care insurance services with a low risk of falls and other conditions, and the investigation of its impact on physical and mental functioning and quality of life, has implications for the rehabilitation of the elderly in the community.

The cost-effectiveness of performing the ARP developed in this study is The Health Technology Assessment (HTA) approach, as comprehensively judged by the principal investigator and the study’s investigators. Further measures to minimize risk and improve cost-effectiveness will be considered.

The appropriateness of carrying out the study will be reviewed by the Tohoku University Hospital Clinical Research Ethics Committee, based on ethical, scientific and medical relevance, and approval will be obtained from the head of the institution.

3 Selection Policy for Research Subjects

3.1 Inclusion criteria

(1) Nursing care level 1 to 3

(2) Those who do not engage in regular aerobic exercise or strength training

(3) Those who has provided an explanation of the research and obtained written consent to participate in the research

(4) Age: 65 years and older

(5) Sex: Male and Female

(6) Users of small-scale, multi-functional in-home care facilities in Adachi-ku, Tokyo who live in the area

(7) Users who have not received compensation

3.2 Exclusion criteria

■Exclusions to influence the safety assessment

(1) Persons with severe neuromuscular, cerebrovascular, or musculoskeletal diseases

(2) Those with medical conditions that have a significant impact on exercise, such as severe cancer or myocardial infarction

(3) Those who are medically prohibited from exercising

■Exclusions from a risk/benefit balance perspective

(1) Patients with severe dementia or psychiatric disorders who are deemed to be unable to participate in the study

(2) Those who have difficulty using an activity meter

Severe dementia or severe mental illness is excluded when the physician determines that the dementia or mental illness prevents the physician from performing an ARP or test item or threatens to exacerbate the cognitive symptoms or mental illness. In this case, the MMSE, one of the secondary endpoints described below, is used. If ARP is initiated within 2 weeks after this time, the MMSE value at this time is used as the baseline (0 month) MMSE value.

4 Research method, period

4.1 Research design

(1) Research design

Control: ARP controlled (ARP Intervention group / control group)

Blinding: non-blinded

Comparison: Randomized Parallel Group Comparison, Intra-subject pre/post comparisons

(2) Rationale for setting up the research design

The test was designed to demonstrate the superiority of ARP.

4.2 Details of the treatment and intervention plan

Explain the study to subjects using a small multifunctional care facility that has received permission to conduct the ARP and confirm that they are willing to consent. Explain the eligibility and exclusion criteria to all subjects who have given consent up to two weeks prior to the intervention and confirm that they meet each of the criteria. Randomly classify subjects into Group A and Group B.

Small-scale multifunctional at-home care in Adachi-ku

1	NPO Kuma House	Koguma	Shikahama 3-17-23	03-3854-4121
2	Sukoyaka Wekfare	Yorimichi-no-ie	Yanagihara 1-29-16	03-5284-2165
3	Care Service Tomo Co., Ltd.	Tomo-no-ie	Hanahata 1-23-13	03-5831-6645
4	Autoso Co., Ltd.	Jasmine-no-ie	Nishiarai 7-10-13	03-5647-9111
5	Human Service Co., Ltd.	Hohoemi-no-ie	Iko 1-6-22	03-5838-2797
6	NPO Platinum Club	Smile Platinum	Ayase 7-4-5	03-5856-1850
7	Mimoza Co., Ltd.	Mimoza Senju-Sakuragi	Senju Sakuragi 2-14-10	03-5284-3828
8	Autoso	Jasmine Ougi	Ougi 1-31-32	03-6807-1278
9	Care Service Tomo Co., Ltd.	Aoi-no-ie	Aoi 2-16-8 1F	03-6807-2613
10	Autoso Co., Ltd.	Jasmine Hanahata	Hanahata 3-5-17	03-5856-7032
11	Conffort Co., Ltd.	Conffort Erva Ayase	Ayase 2-14-14	03-5629-3080
12	Ookuma	Shimanami	Tsubaki 2-22-2	03-5856-9537
13	Sonoda-kai	Nursing home Sonoda	Hokima 1-29-12	03-5831-0400

As a manager of the Adachi Ward Multi-Functional Service Liaison Group, which consists of the above 13 facilities, he liaises and coordinates with the administrators and planners of each facility regarding the implementation of the study.

The ARP is a community rehabilitation program with weekly participation, one cycle of four weeks.

The first week, once a week, I ride the bus for about 10-20 minutes to go shopping for park cleanup and flower bed maintenance needs. It takes three hours to travel and shop. For the next three weeks, they will spend one hour once a week at a neighborhood park to clean up and manage their flower beds. The park is located in the neighborhood of the nursing home (less than 500 meters away) and is about 1,000m^2 in area with some rolling terrain. Walk to the park (cane and silver car available). Cleanup activities include carrying tongs and garbage bags and moving freely around the park to pick up trash. To maintain the flower beds, digging the soil, planting flowering plants and watering them with a hose. These activities are carried out under Adachi Ward's "Self-management of Parks" and "Self-management of Flowerbeds" systems.

Do this for three cycles (12 weeks). On other days, the participants live as normal.

Group A Perform the above ARP once a week.

Group B While the intervention group is performing the ARP, they are asked to choose from three different activities, mainly seated, to watch TV, colouring books, and folding laundry, as their usual care.

Physical and mental functioning will be assessed at the study site for each of the two groups categorized. The primary efficacy endpoints will be the weekly activity (number of steps) and TUG (Timed Up and Go). Secondary endpoints will include the Barthel Index (BI), Functional Independence Measure (FIM), Mini Mental State Examination (MMSE), weekly activity (MET/hour), maximum 10-meter walk and grip strength. Group A will then receive ARP and Group B will receive usual care for 12 weeks. After 4 weeks, physical and mental functioning will be assessed at 12 weeks.

All evaluations, including this program, will be conducted by the principal investigator or research co-investigator.

(1) Test Equipment

An ACOS AM500N pedometer will be used to measure the number of steps and activity. The pedometer is loaned from the laboratory and will be worn for seven consecutive days.

A T.K.K. 5401 Grip-D digital grip strength meter from Takei Instrument Industry will be used to measure grip strength.

(2) Level of intervention

Explain the study to the subject and confirm that he or she agrees to the study. If consent is obtained, the eligibility and exclusion criteria will be explained to the subject at least two weeks prior to the exercise intervention and we will confirm that each criterion is met.

(3) Criteria for changing the level of intervention

In this study, the content and dosage for all interventions will not change until the study is completed. If a

subject misses one or more ARPs due to urgent needs, the next intervention will proceed as specified, even if not all interventions can be implemented. In the unlikely event that the ARP is unsustainable, the subject in question will be considered an omission.

(4) Cancellation and completion criteria.

During the implementation of the exercise, the research subjects were asked to Refusal of research due to serious adverse events or adverse events or for other reasons not related to the adverse event, including relocation or change in long-term care insurance services; and Discontinue the intervention immediately. If the study subject completes the ARP intervention without the occurrence of any adverse events, the intervention will be considered complete.

(5) Personal information.

Management and anonymization of personal information related to this research will be performed by Adachi Ward Multi-Functional Service.

Yoshihiko Baba, Personal Information Manager (Adachi Ward Multifunctional Service Liaison Committee)

4.3 Combination therapy

(1) Acceptable concomitant therapies: medications or nutritional therapy used prior to the study. Aerobic exercise necessary for daily living, such as walking and climbing stairs.

(2) Unacceptable concomitant therapies: aerobic exercise or strength training other than ARP.

4.4 Post-treatment

No ARP will be conducted after the ARP is cancelled.

After the ARP is terminated, the ARP shall continue to be conducted.

4.5 Inspection schedule

Testing will be done before the study, 4 weeks after the study and 12 weeks after the study.

4.6 Research period

Research period: November 2017 (after Ethics Committee approval) - September 2021

Participation Schedule for Participants: November 2017（After approval）～ February 2018

Month	Week	Group A	Group B
		ARP intervention	control
November	4	Evaluation (pre)	
December to February	1	Bus trip,. shopping	Usual care at service provider
	2	Cleaning the park	Usual care at service provider
	3	Cleaning the park	Usual care at service provider
	4	Cleaning the park	Usual care at service provider
March	1	Evaluation (12 weeks)	

'Usual care in the office' means that while the ARP intervention group is performing ARP, they are asked to choose from three main activities: watching TV in a seated position, colouring books, and folding laundry.

5 Assessment of Adverse Events

An adverse event is defined as any unfavorable, unavoidable, or unintended consequence to the research subjects and surgeons who conducted the research. or unintended disease or disorder and its signs (including abnormal laboratory values); and No causal relationship to the study drug/instrument will be established.

Adverse events will be collected from the conduct of the study to the end of the individual study subjects' observation period or at the time of discontinuation.

5.1 Obtaining Information

(1) In the event of a serious adverse event/failure, the investigator(s) will take appropriate action and report to the principal investigator of the institution.

(2) The principal investigator of the institution will confirm the following with the investigators

Items to be confirmed by the principal investigator to the researcher

1. Harmful / Unsuitable
2. Severity Classification [1)]
3. Reiteration[2)] Reasons for judging it to be serious
4. Predictability (unknown and known)[3)]
5. Causal relationship with the intervention (study drug/instrument)
6. History of the event/failure (date of onset, course, outcome, etc.)
7. Information about the subject's identification (initials, age and gender)

[1)]Severity Classification

Assessed according to:

National Cancer Institute Common Terminology Criteria for Adverse Events

(NCI CTCAE v4.0 : http://www.jcog.jp/doctor/tool/CTCAEv4J_20150310.pdf)

If there are no items that fall under the NCI CTCAE classification, the following "Criteria for Classification of Severity of Adverse Events" should be used as a guide.

Severity Classification (NCI CTCAE Grade)	Criteria
light （Grade 1）	No symptoms or mild symptoms. Clinical or laboratory findings only. Does not require treatment.
moderate （Grade 2）	Requires minimal/local/non-invasive treatment. Restrictions on activities of daily living other than age-appropriate personal activities*.
Severe （Grade 3）	Serious or medically significant, but not immediately life-threatening. Requires hospitalization or extended hospitalization. Incapacity/inability to perform activities. Limitations in activities of daily living around you**.
Very severe （Grade 4）	Life-threatening. Requires immediate action.
Death （Grade 5）	Death from adverse events (AEs).

*Activities of daily living outside of immediate surroundings（instrumental ADL）

: Preparing meals, shopping for household items and clothing, using the phone, managing money, etc.

**Activities of daily living（self care ADL）

: Able to bathe, dress and undress, consume food, use the toilet, take medication, and not be bedridden.

[2)]Definition of critical illness

Items to be confirmed by the principal investigator to the researcher

- something that will lead to death
- A threat to life.
- Those that require hospitalization or extended hospitalization for treatment
- Any permanent or significant disability or dysfunction
- A congenital anomaly in the offspring.

Hospitalizations specified in the research protocol, hospitalizations for the sole purpose of performing therapies or tests that have been scheduled since before the research (before consent was obtained) during the research (e.g., scheduled surgeries and tests), or hospitalizations other than for the purpose of treatment or tests associated with an adverse event (e.g., medical examinations) will not be treated as serious adverse events.

3) Definition of predictability

Items to be confirmed by the principal investigator to the researcher

Unpredictable (unknown)
The occurrence of the event, or the tendency of the event, such as the number of occurrences, frequency of occurrences, or conditions of occurrence, cannot be predicted from the official documents (e.g., appendices and papers) for the study drug/instrument in question.
Predictable (known)
The occurrence of the event, or the tendency of the event, such as the number of occurrences, frequency, and conditions of occurrence, can be predicted from the official documentation of the study drug/instrument in question (ibid.).

5.2 Description of Adverse Events

For all adverse events that occur, the investigators and others will include in the case report the name of the adverse event, the extent (serious, non-serious), the reason for the determination of seriousness, the date of onset, the date of the outcome, the treatment, the outcome (recovery, lightening, recovery but with sequelae, unrecovered, death), the causal relationship to the study, and comments (e.g., causal relationship and reasons for determination).

This study involves outdoor activities that may pose a health hazard. On the day of the intervention, body temperature and blood pressure will be measured in advance and the physician/nurse at the study site will determine whether or not to participate.

Dizziness, pallor, profuse sweating, shortness of breath, and abnormalities in blood pressure and heart rate due to changes in circulatory dynamics are possible to manifest. In addition, musculoskeletal disorders of arthralgia and myalgia in the lower extremities may occur. If adverse events occur, physicians and nurses will respond appropriately.

6 Response to serious adverse events/failures (including the range of adverse events to be reported to the head of the institution)

6.1 Response to Adverse Events/Failures

(1) In the event of an adverse event/defect, the Investigator will take appropriate action and endeavor to investigate the cause of the event while paying attention to ensuring the safety of the research subjects.

(2) In principle, the investigators will keep the symptoms or abnormal changes in clinical laboratory values that occur until the event in question has disappeared or has returned to the state before the research began. or until it is determined that the outcome is not clinically problematic, continue to follow up to the extent possible.

(3) If the investigator or others determine that no follow-up is necessary, such as if the unrecovered adverse event/failure is a non-reversible event at the end of the study Follow-up will be terminated at the end of the study

subject's study, and the reason for the decision not to follow-up will be noted in the comments section of the case report form.

6.2 Report to the head of the institution and the principal investigator (principal investigator)

(1) The principal investigator of the institution shall report the occurrence of a serious adverse event/failure to the head of the institution within the following timeframe from the time he or she becomes aware of it. The report should be made in the form of "(Form 9) Report on Serious Adverse Events"*.

*Download the Clinical Research Form `http://www.med.tohoku.ac.jp/public/rinri_d.html`

	light/moderate/severe (Grade 1/2/3)			very severe (Grade 4)		death		Other medically important states
	Unpredictable (Known)	Unpredeitable (Unknown)		Predictable (Known)	Unpredictable (Unknown)	Predictable (Known)	Unpredictable (Unknown)	
	Hospitalized No/Yes	Hospitalized No	Hospitalized Yes					
No causation								
	Report<br>N/A	Report<br>N/A	First report Within 10 days<br>Additional report : As required	Primary report: within 72 hours<br>Secondary report: within 7 days<br>Additional report: as required				
			Minister Target Grade 3 only		Minister Target		Minister Target	
Causation								
	Report<br>N/A	Report<br>N/A	First report : 10 日以内<br>Additional report : As required	Primary report: within 72 hours<br>Secondary report: within 7 days<br>Additional report: as required				

*Only during the intervention or within 30 days of the last protocol intervention date

6.3 Report to the Minister of Health, Labour and Welfare (administrative authorities)

The head of the institution shall be responsible for any adverse event/failure that is "unpredictable" and "of undeniable causation" and "severe*". Promptly report to the Minister of Health, Labour and Welfare, through the Director General, by means of a **(Reference Form 1) Unpredictable Serious Adverse Event/Defect Report**.

*Severe
Death, Very Severe （Grade4），
Light, Moderate, Severe (Grade 1/2/3) that requires hospitalization or extended hospitalization for treatment

6.4 Response to the Efficacy and Safety Evaluation Committee

In the case of research with an Efficacy and Safety Evaluation Committee, the principal investigator will ask the Efficacy and Safety Evaluation Committee to review the following

1. Evaluation of Information
2. Whether the plan needs to be changed
3. Whether the research can be continued
4. Other (e.g., suspending new enrollment, revising the informed consent document, re-consenting to other subjects, etc.)

6.5 Release of information

(1) The head of the institution will respond to unpredictable and serious adverse events and publish the results.

(2) If the head of the research institution becomes aware of any serious nonconformity to the Integrated Guidelines with regard to medical research on human subjects that is currently being conducted or has been conducted in the past, the head of the research institution shall promptly obtain the opinion of the Ethics Committee, take the necessary action, report the status and results of that action to the Minister of Health, Labour and Welfare, etc., and make a public announcement.

7 Survey items and methods

7.1 Primary outcomes

Assessments will be done before the study, 4 weeks after the study and 12 weeks after the study.

■Physical activity (number of steps) Current pedometers are highly accurate and reliable.[16] Epidemiological studies of healthy older adults using a pedometer show that walking 4,000 steps per day is effective in maintaining mental health and 7,000 steps per day is effective in maintaining physical health. [5] In addition, the Ministry of Health, Labour and Welfare (MHLW) has set the threshold for physical activity for the elderly at 10 METs/hour/week in the Physical Activity Standards 2013, [17] The equivalent number of steps is stated to be 4376 steps per day. [18]

An intervention study found that an 8-week mall walking program in healthy older adults (66.38 $\pm$ 8.13 years old, 52 participants) increased the number of daily steps from 5,055 $\pm$ 1,374 to 5,969 $\pm$ 1,543 steps[19].

Before the study, 4 weeks after the study, and 12 weeks after the study, the number of steps for 7 consecutive days will be measured with a triaxial accelerometer.

■Timed Up and Go (TUG) measures the time to stand from a chair sitting position on a chair at a height of about 46 cm, walk 3m, turn, walk back and sit down. A stopwatch is used to measure the sequence of movements [20]. TUG has high intra- and inter-examiner reliability, and is closely related to lower extremity muscle strength, balance, gait ability, and daily function. It can also be used as a fall prediction test. [21]

TUG improved from 12.4 seconds to 8.02 seconds after 3 months of outpatient rehabilitation for patients who had undergone knee arthroplasty [22] . In Japan, it has been confirmed that it is useful for the elderly and frail elderly living in the community. [23] [24] .

TUG will be measured before the study, 4 weeks after the study and 12 weeks after the study.

7.2 Secondary outcomes

Secondary outcomes will be measured before the study, 4 weeks after the study and 12 weeks after the study.

7.2.1 physical function assessment

■Physical activity (Met hour)) Activity meters are becoming more accurate, with triaxial accelerometers replacing single-axis accelerometers. For example, Yamada et al. [25] compared uniaxial and triaxial accelerometers to the double-labeled water method and argued for the importance of measuring low intensity exercise with triaxial ac-

celerometers. On the other hand, Park et al [26] compared Kenz Lifecorder EX, Actimarker and Active Style Pro and found that while they were accurate at normal speeds (75 m/min), at lower speeds (55 m/min), the accuracy varied by model. Older people have lower total steps, total energy expenditure, etc., and especially less physical activity above 2.2 METs compared to young people. [27] However, accelerometers are not intended for the elderly and need to be handled with caution. [28]

Activity (MET/hour) over seven consecutive days is measured with a triaxial accelerometer.

■10 m Walk Test Bowden et al. [29] found that based on gait speed, <0.4 m/s is "potentially independent of indoor walking" (household), 0.4 m/s to 0.8 m/s is "gait independence to a limited extent" (limited), and >0.8 m/s is "gait independence" (full). Even in determining sarcopenia, 0.8 m/s is the standard. [30] [31]. In addition, walking speed is a better predictor of care dependence than grip strength, which will be discussed below, and open-eyed one-legged standing, which will not be measured in this study. [32] In Japan, traffic signals are often set at a walking speed of 1.0 m/s, and it has been proposed that 1.0 m/s is a suitable standard.

■Grip strength is correlated with overall muscle mass and is an indicator of prognosis/ [33] The most commonly used method of measuring grip strength is to use a Smedley-type grip strength meter to measure the grip strength of both hands twice alternately and adopt the highest value.

■Barthel Index (BI) It was developed by Mahoney et al. in 1965 and was commonly used in ADL assessment scales until the FIM, discussed below, was adopted. It assesses 10 items such as eating, transferring, dressing, toileting, bathing, moving, stair climbing, changing, defecation self-control, and urination self-control with several levels of independence and partial assistance, respectively. Although BI is an excellent inter-examiner reproducible assessment method, it has a weakness that makes it difficult to capture improvements in symptoms.

■Functional Independence Measure (FIM) ADL rating scale translated as Functional Independence Assessment [34]. There are 13 motor items and 5 cognitive items, each of which is rated from 1 to 7 points. A score of 126 points is given when the ADL is independent and 18 points for all assistance. It is said to be the most reliable and valid of all ADL assessment scales.

7.2.2 Psychological function assessment

■Mini-Mental State Examination (MMSE) The MMSE is a simple scale to test the severity of dementia. It consists of 11 items measuring abilities such as disorientation, memory, calculation, cognition, movement and graphic description. A score of 21 to 26 indicates mild dementia, 15 to 20 indicates mild to moderate dementia, 10 to 14 indicates moderate dementia and less than 10 indicates severe dementia. The lower the total score, the more severe the cognitive impairment. [35]

■Short Form 8 (SF-8) is an abbreviated version of the SF-36, a widely used health-related quality of life (HRQOL) scale that measures (1) physical functioning (2) daily role functioning (body) (3) body pain (4) overall health (5) vitality (6) social life functioning (7) daily role Function (spirit), (8) mental health [36]. It consists of a common concept, HRQOL, which allows for the measurement of health-related quality of life for various diseases and allows for comparison of quality of life between patients with different diseases.

■EuroQOL 5 Dimension 3 L (EuroQOL-5D-3L) There are many ways to measure a patient's quality of life, but EuroQOL-5D-3L [?] is so easy to use, and for so many diseases, that it can quantify quality of life from the highest (1.0) to the lowest (0.0) in five questions [37]. It is often used to calculate health economic evaluation.

While EuroQOL-5D-3L has three levels of each question, the EuroQOL-5D-5L, which has five levels of [38] that have been developed.

7.3 Interim Judgment of Evaluation

Interim judgment of evaluation will not be conducted.

8 Registration and Allocation

8.1 Registration

(1) Registration Process

After enrollment, the eligibility and exclusion criteria are examined from the basic information of the establishments, and those eligible that meet the criteria are randomly assigned to Group A and Group B.

(2) Notes on Registration

Enrollment after the study has begun is not acceptable.

In the event that an error or duplicate registration is found, the research secretariat will be notified immediately.

8.2 Allocation

(1) Allocation methods and allocation adjustment factors

The method of random assignment (randomization) will be stratified randomization.

The allocation adjustment factors will be age, gender, primary disease, and level of care required.

The details of the random assignment procedure will not be disclosed to the researchers, etc., but will be retained by the person in charge of allocation.

(2) Basis for the establishment of the allocation adjustment factors

Age, gender, and primary disease were considered to affect activity (steps) and TUG as well as the level of care required.

9 Expected number of cases, and its reasons

9.1 Expected number of cases

The planned caseload is 60 in Group A and 60 in Group B, for a total of 120 patients.

9.2 Reasons

The sample size was calculated with reference to previous studies. Significant post-intervention improvements in activity (steps) and TUG emerged ($\mu > \mu_0$), with significance levels ($\alpha = 0.05$) and effect sizes ($\delta = 0.50$) and test power ($1 - \beta = 0.80$) as Assuming a two-tailed test with a corresponding t-test as a randomized controlled trial, the number of subjects in each group would be 50. Assuming that about 10% of the participants would drop out, each group would have 60 participants each, for a total of 120 participants.

9.3 Prospective Research Subjects

The sample size for this study will be 100 participants. Assuming that about 10

The maximum number of people registered in the small-scale multifunctional in-home care business is 29, and there are 13 businesses in Adachi-ku, Tokyo. There are 13 facilities in Adachi Ward, Tokyo, and each has about 20 users, so the total number of users is thought to be about 260. The users are generally in the range of care needs 1-3.

10 Statistics

10.1 Statistical methods

Group A (ARP intervention group) and Group B (control group) will be included in the analysis of this study. All measures will be checked for normality by the Shapiro-Wilk test. Subsequently, mean comparisons of parametric or non-parametric methods according to the presence or absence of normality in the comparisons before and after the

intervention within the groups will be performed, and Regression analysis and analysis of covariance will be performed for comparisons between groups. The test is a two-tailed test, with $p<0.05$ being the significance level.

10.2 Interim analysis and early termination of the study

No interim analysis will be performed.

11 Data management and self-assessment methods

11.1 Preparation of Case Report Forms (CRFs)

Entries and corrections in the CRF should be made by the researcher. The researcher will complete the CRF for each subject as soon as possible after the completion of each study. They will follow the instructions for filling out the CRF, as outlined in the Guide to Writing Case Reports. When preparing the CRF, make sure that it can be read and understood by a third party, be careful not to make any mistakes, and be careful not to make any mistakes in the entry of the CRF. Note that the data should be recorded in legible handwriting and not in pencil. The research collaborator may transcribe from the source material to the CRF if the source material exists and its objectivity can be assured.

11.2 CRF self-inspection

(1) The researcher shall check the consistency between the CRF and the source documents (e.g., raw data).

(2) If there are any discrepancies between the CRF and the source documents, the investigators will prepare a record explaining the reasons for the discrepancies.

(3) The principal investigator will check the contents of the CRF and sign the CRF with his or her name and seal or signature after confirming the contents.

11.3 Sending and storing of CRFs

The principal investigator of the research organization shall submit the original CRF to the research office in accordance with the established procedure. The principal investigator must submit the CRF to the following address

(CRF submission address)
Research Secretariat
Address: 1-1 Seiryo-cho, Aoba-ku, Sendai-shi, Miyagi 980-8574
Department of Rehabilitation Science and Internal Medicine, Tohoku University Graduate School of Medicine
Phone: 022-717-7353

11.4 CRF Correction Procedure

If the CRF is to be amended, the principal investigator of the institution will submit a record of the change or amendment to the CRF in accordance with established procedures and retain a copy.

12 Procedures for obtaining informed consent

12.1 Explanation to Research Subjects

The researcher(s) shall provide the subject(s) with a letter of explanation approved by the institution prior to registration, explaining the following

Description of explanatory documents

1. The name of the research and a statement that the head of the institution has given permission to conduct the

research

2. Research Institute, Principal Investigator
3. Purpose and significance of the research
4. Research methods, duration
5. Reasons for being selected as a research subject
6. The burdens, projected risks and benefits to research subjects
7. A statement that consent to the implementation and continuation of research can be withdrawn at any time.
8. A statement that no research subject or other person will be subjected to disadvantageous treatment due to non-consent or withdrawal of consent to conduct or continue research
9. How to publish information about your research
10. How to obtain and access materials related to research protocols and research methods in response to requests from research subjects and others to the extent that it does not interfere with protecting the personal information of other research subjects and ensuring the originality of the research.
11. Handling of personal information, etc. (including the method for anonymizing it)
12. How to store and dispose of samples and information
13. Conflict of interest (sources of funding for research, possible conflicts of interest, relationships with researchers and other relevant organizations)
14. Responses to consultations with research subjects and related persons
15. Details of any financial burden/gratuity paid to research subjects, etc.
16. If the research involves medical treatment beyond the normal course of treatment, matters relating to other treatment methods, etc. Not applicable.
17. If the study involves medical treatment beyond the normal course of treatment, measures related to the provision of medical care after the study is completed Not applicable
18. Treatment of research results (including incidental findings) pertaining to research subjects if the conduct of the research is likely to yield important findings, such as health of the research subject or genetic characteristics that may be passed on to offspring.
19. In the case of invasive research, the existence and content of compensation for health hazards caused by the research
20. If there is a possibility that samples or information obtained from a research subject may be used for future research that is not specified at the time consent is received from the research subject or others, or that may be provided to other research organizations, then it is assumed at the time consent is received.
21. In the case of intervention research involving invasion (except for minor invasion), the monitoring and auditing personnel and ethics committees will have access to samples and information about the research subjects, provided that the confidentiality of the research subjects is preserved.

12.2 Consent

After explaining the research, giving the subject sufficient time to think about it, and confirming that he or she understands the study, we ask him or her to participate in the study. If the research subject agrees to participate in the study, the consent document will be signed by the subject himself or herself.

One copy of the consent document will be kept by the principal investigator at the institution, and one copy will be given to the subject.

13 (Procedures for receiving informed consent from a proxy, etc.)

No informed consent will be obtained from the delegate or others.

14 Procedures for obtaining an informed assent

We do not envisage a situation where you get informed assent.

15 Handling of Personal Information, etc.

15.1 Purpose of Use of Personal Information

In order to obtain the correct results of the research, personal information will be used for the purpose of identifying and investigating individual research subjects not only during the research, but also for a long period of time after the completion of the research, and for the proper management of the information obtained.

15.2 How to use (anonymization method)

Care will be taken to ensure that personal data such as the name, date of birth, address, telephone number, etc., of the enrolled subject cannot be identified by identification or inquiry of the enrollee, or by the subject identification code issued at the time of registration, except in special cases. Special cases refer to the withdrawal of a subject after consent to research or a peripheral request for data, or when an adverse event occurs, and only then will the administrator of the site where the research will be carried out prepare a correspondence list and anonymize the data.

15.3 Safety Management System (Safety Management Measures for Personal Information)

The principal investigator of the research institution will take security control measures to minimize the risk of information leakage when using personal information.

16 Burden on research subjects, anticipated risks (including possible adverse events) and benefits, a comprehensive assessment of these, and measures to minimise the burden and risk.

16.1 Summary of anticipated benefits and disadvantages associated with research participation

(1) Projected Benefits
Group A may improve their motivation and their ability to take steps and balance on a daily basis. Quality of life (QOL) may also improve.
Group B is expected to have no direct benefit.

(2) Anticipated risks and disadvantages
Group A may pose a health risk because it involves outdoor activities. On the day of the intervention, body temperature and blood pressure will be measured in advance and the physician/nurse at the study site will decide whether or not to participate. Dizziness, pallor, profuse sweating, shortness of breath, and abnormalities in blood pressure and heart rate due to changes in circulatory dynamics are possible to manifest. In addition, musculoskeletal disorders of arthralgia and myalgia in the lower extremities may also occur.
Group B is not expected to have any direct adverse effects.
In the event of an adverse event, the physician or nurse will respond appropriately and will not provide financial compensation.

17 Methods for Storage and Disposal of Samples and Information

17.1 Sample preservation

The principal investigator shall preserve the samples and information as follows

Keepers	Samples and information	Preservation period
Investigators	Samples and information obtained from the human body<br>Documents or source materials pertaining to research to be preserved at the research institution<br>Procedures, etc.<br>Register of Members	5 years from the study end date<br><br>Three years from the date of publication of the re<br>(whichever is later.)

Note that no samples will be obtained from the human body.

17.2 Abandonment

The principal investigator and the principal investigator of the research institution will anonymize samples and information obtained from human subjects when they are disposed of.

18 Conflicts of interest regarding research sources, conflicts of interest relating to research at research institutions, and individual earnings, and conflicts of interest relating to research by researchers and others

This research is funded by a management grant. This study was carried out with an operating grant. This study will be conducted on users of small-scale multifunctional in-home care facilities in Adachi-ku, mainly Jasmin Ougi, a small-scale multifunctional in-home care facility (operated by AUTSOO Co., Ltd.), where the research co-researcher works.

Conflicts of interest in this research will be documented in the research protocol and in the disclosure documents, based on the fact that the World Medical Association Declaration of Helsinki and the Ethical Guidelines for Medical Research Involving Human Subjects (Ministry of Education, Culture, Sports, Science and Technology, Ministry of Health, Labour and Welfare) require sufficient explanation of funding, sponsorship, and conflicts of interest to research subjects and to be documented in the research protocol. Conflicts of interest of Tohoku University researchers and others shall be managed by the Conflict of Interest Management Committee of Tohoku University.

Regarding the "self-management of the park," Jasmine Ougi receives compensation from Adachi Ward. Users who receive this compensation are excluded from the study.

19 Intellectual Property

The results, data and intellectual property rights obtained in this study will belong to Tohoku University. The specific handling and distribution of intellectual property rights will be decided through discussion. Whether the intellectual property rights belong to an individual or a research institution will be decided according to the agreement of the research institution to which the researcher belongs.

20 How to publish information about your research

20.1 Registering a Research Plan

The principal investigator will register the study abstract in the Clinical Trials Registration System (University Hospitals Medical Information Network, UMIN-CTR) and update it as necessary when the research protocol is changed or the study progresses.

`http://www.umin.ac.jp/ctr/index-j.htm`

20.2 Registration of Research Results

The principal investigator will register the results of the research in a public database after the research is completed. However, matters that are not open to the public in order to protect the human rights of research subjects and others, the human rights of researchers and others involved, and intellectual property, and matters that are approved by the head of the research institution in response to an ethics committee's opinion because they would significantly interfere with the research from the perspective of protecting personal information, shall not be open to the public.

20.3 Publication of Research Results

The principal investigator will take measures to protect the personal information of the research subjects, and publish the results of the research in a medical journal or other media without delay after the completion of the research. In the case of final publication of the results, the principal investigator will report to the head of the research organization without delay.

21 Content and method of reporting to the head of the research institution

The principal investigator shall report the following to the head of the institution via **(Form 8) Report on the Progress of the Study**.

- Progress of Research
- The occurrence of adverse events associated with the conduct of research
- Summary of Research Completion/Cancellation and Results

22 Responding to consultations, etc., from research subjects and other related parties

For general research inquiries (contact information)
Yoshihiko Baba

Department of Rehabilitation Science and Internal Medicine, Tohoku University Graduate School of Medicine
Seiryo-cho 1-1, Aoba-ku, Sendai, 980-8574
TEL: 022-717-7353 FAX: 022-717-7355
Jasmine Ougi (Small Multifunctional At-home Care)
Ougi 1-31-32, Adachi-ku, Tokyo 123-0873
TEL: 03-6807-1278 FAX: 03-6807-1279

Contact for inquiries about this Privacy Policy
Yoshihiko Baba

Department of Rehabilitation Science and Internal Medicine, Tohoku University Graduate School of Medicine
Seiryo-cho 1-1, Aoba-ku, Sendai, 980-8574
TEL: 022-717-7353 FAX: 022-717-7355
Jasmine Ougi (Small Multifunctional At-home Care)
Ougi 1-31-32, Adachi-ku, Tokyo 123-0873
TEL: 03-6807-1278 FAX: 03-6807-1279

23 How to determine the satisfaction of all requirements when conducting research under emergency conditions

- The subject of the study is in immediate and obvious danger of death.
- Not enough effect in usual practice, and intervention studies have a good chance of averting life threats for study subjects
- A minimum amount of burden and risk to the research subject resulting from the research
- Not being able to immediately contact the proxy

24 If there are any financial burdens or rewards to be paid to the research subjects or others, please describe them in detail.

Regarding the "self-management of the park," Jasmine Ougi receives compensation from Adachi Ward. Users who receive this compensation are excluded from the study.

25 In the case of invasive research, whether and what compensation is available for health hazards caused by the research

If health hazards occur to a research subject as a result of the conduct of the research, the institution will take treatment and other necessary measures. There will be no monetary compensation.

The subject will bear any out-of-pocket medical expenses.

26 Description of work and supervision of contractors

We will not outsource the work of research.

27 The possibility that the sample/information may be used for future research that is not specified at the time the consent is received/probable to be provided to other institutions, if any, and what is anticipated at the time the consent is received

There will be no secondary use of the data obtained in this study.

28 Monitoring System

The principal investigator will ask the data controller and monitoring personnel to monitor the study to ensure that the study is being conducted safely and in accordance with the research protocol and that data are being collected accurately.

The principal investigator will prepare a monitoring plan, and the data controller and the monitoring staff will carry out the monitoring based on the monitoring plan.

The data controller and the monitoring personnel will submit a monitoring report to the principal investigator.

① Monitoring refers to onsite monitoring, including verification against the source documents, by monitoring staff who visit the facility based on the data entered in the CRF by the data controller.

② Since the purpose of monitoring is to improve the scientific ethics of the study by providing feedback on problems and is not intended to expose problems in the study or the institution, the principal investigator will review the

monitoring reports and share information about the problems noted with the principal investigator, researchers, and others at the institution in an effort to improve the study. The data controller and monitoring personnel will mainly monitor for the presence or absence of the following

violation

An event that was not conducted in accordance with the research protocol and that falls into more than one of the following categories shall be considered a "violation

substantially affect the assessment of the test endpoints.

Deliberate or systematic

Significant degree of danger or deviation

deviation

An event that was not performed in accordance with the research protocol is considered a "deviation".

However, if it is determined to be medically dangerous by following the research protocol and in accordance with the medical judgment of the researcher,

any change in treatment should be treated as a "clinically reasonable deviation".

acceptable deviation

An acceptable deviation from the research proposal shall be referred to as an "acceptable deviation".

③ If clinically reasonable deviations are frequent, consider revising the research protocol.

④ Setting an acceptable range of deviations is not always desirable because systematic bias within the acceptable range may affect the results of the study. The decision should be made after taking into account the nature of the study and the degree of familiarity of the researcher or other person with the study.

⑤ In principle, the details of the violation should be described in the publication of the paper. If there are many specific deviations, it is preferable to list the details of the deviation.

29 Changes to the Research Proposal

When amending a research protocol, the principal investigator and the principal investigator of the institution will obtain the approval of the head of the institution after review by the ethics committee. Changes to the content of a research protocol will be handled in two ways: revision and revision. Other additions to the supplementary explanations that do not constitute changes to the research protocol will be distinguished as memorandums.

(1) Amendment

Changes to the research protocol that may increase the risk to research subjects or affect the primary endpoint. Requires the approval of each institution. The following are applicable.

- Changes that increase the burden on subjects (more invasive blood collection, testing, etc.)
- Change in exclusion criteria etc. due to the occurrence of serious side effects
- Changes to the effectiveness and safety evaluation method
- Change in the number of cases

(2) Revision

Changes to the research protocol that are not likely to increase the risk to research subjects and that do not affect the primary endpoint. Requires approval of each institution. This applies to the following cases

- Changes that do not increase the burden on subjects (change in timing of testing)
- Change of the study period

- Change of Researcher

(3) Memorandum

A supplemental explanation of the research protocol distributed by the principal investigator to those involved in the research, not to change the content of the research protocol, but to reduce variation in the interpretation of the text, especially to draw attention to it.

30 Research System

30.1 The name of the research institution and the name of the principal investigator

Principal investigator

Prof. Masahiro Kohzuki
Department of Rehabilitation Science and Internal Medicine, Tohoku University Graduate School of Medicine
Seiryo-cho 1-1, Aoba-ku, Sendai, 980-8574
TEL 022-717-7351 FAX 022-717-7355
E-mail: kohzuki@med.tohoku.ac.jp

30.2 Collaborative Research Institute

(1) Collaborative Research Institute: None

30.3 Roles and responsibilities of research secretariats, collaborating institutions and principal investigators

Research Bureau

Department of Rehabilitation Science and Internal Medicine, Tohoku University Graduate School of Medicine
Seiryo-cho 1-1, Aoba-ku, Sendai, 980-8574
TEL 022-717-7353 FAX 022-717-7355
E-mail: babayoshihiko@mac.com

(2) Principal Investigator

Department of Rehabilitation Science and Internal Medicine, Tohoku University Graduate School of Medicine
Prof. Masahiro Kohzuki

(3) Research contributors (Tohoku University)

Department of Rehabilitation Science and Internal Medicine, Tohoku University Graduate School of Medicine
Assistant Yosuke Izoe
Doctor Chika Ooyama
Associate Professor Yasushi Tazawa
Graduate student Yoshihiko Baba

30.4 Efficacy and Safety Evaluation Committee

1 Department of Rehabilitation, Faculty of Health Sciences, Tohoku Fukushi University
Associate Professor Kazuaki Iokawa
2 Department of Health Care Administration, Faculty of Health Sciences, Tohoku Fukushi University
ssociate Professor Takayuki Kawamura

30.5 Statistical Analysis, Data Center

(1) Head of Statistical Analysis

Department of Rehabilitation Science and Internal Medicine, Tohoku University Graduate School of Medicine
Graduate student Yoshihiko Baba
Seiryo-cho 1-1, Aoba-ku, Sendai, 980-8574
TEL 022-717-7353 FAX 022-717-7355

(2) Data controller

Department of Rehabilitation Science and Internal Medicine, Tohoku University Graduate School of Medicine
Prof. Masahiro Kohzuki

(3) Monitoring personnel

Department of Rehabilitation Science and Internal Medicine, Tohoku University Graduate School of Medicine
Assistant Tamao Takahashi

30.6 Contact for Research Inquiries

(1) How to register research subjects (participants) Graduate student Yoshihiko Baba

Department of Rehabilitation Science and Internal Medicine, Tohoku University Graduate School of Medicine
Seiryo-cho 1-1, Aoba-ku, Sendai, 980-8574
TEL 022-717-7353 FAX 022-717-7355
E-mail: babayoshihiko@mac.com

(2) How to respond to adverse events Prof. Masahiro Kohzuki

Department of Rehabilitation Science and Internal Medicine, Tohoku University Graduate School of Medicine
Seiryo-cho 1-1, Aoba-ku, Sendai, 980-8574
TEL 022-717-7351 FAX 022-717-7355
E-mail: kohzuki@med.tohoku.ac.jp

31 引用文献

参考文献

[1] Hirohisa Imai, Yoshinori Fujii, Yoshiharu Fukuda, Hiroyuki Nakao, and Yuichiro Yahata. Health-related quality of life and beneficiaries of long-term care insurance in Japan. *Health Policy*, 85(3):349–355, 2008.

[2] Nanako Tamiya, Haruko Noguchi, Akihiro Nishi, Michael R Reich, Naoki Ikegami, Hideki Hashimoto, Kenji Shibuya, Ichiro Kawachi, and John Creighton Campbell. Population ageing and wellbeing: lessons from Japan's long-term care insurance policy. *The Lancet*, 378(9797):1183–1192, 2011.

[3] MHLW. New rehabilitation approches for the elderly in community: Report (in Japanese), 2015.

[4] 金谷さとみ. 地域における生活機能向上のための理学療法. **理学療法ジャーナル**, 38(7):529–535, 2004.

[5] Yukitoshi Aoyagi and Roy J Shephard. Habitual physical activity and health in the elderly: the nakanojo study. *Geriatrics & Gerontology International*, 10(s1):S236–S243, 2010.

[6] Satoru Kanemori, Yuko Kai, Jun Ida, Katsunori Kondo, Ichiro Kawachi, Hiroshi Hirai, Kokoro Shirai, Yoshiki Ishikawa, and Kayo Suzuki. Social participation and the prevention of functional disability in older Japanese: the JAGES Cohort Study. *PLoS ONE*, 9(6):e99638, 2014.

[7] 入江多津子. リハビリテーションとケアマネジメント. **総合リハビリテーション**, 38(6):519–525, 2010.

[8] 山永裕明, 野尻晋一, 中西亮二, 桂賢一, 渡邊進, and 米満弘之. 介護保険下の脳卒中維持期リハビリテーション. **リハビリテーション医学**, 42(1):58–71, 2005.

[9] Takako Itsukaichi, Yoshimi Sukukamo, and Shin-Ichi Izumi. Factors influeincing the planning of home-based rehabilitation services by care managers. *Japanese Journal of Comprehensive Rehabilitation Science*, 4:39–46, 2013.

[10] 須貝佑一 and 小林奈美. 施設における痴呆高齢者の転倒・転落事故の発生状況と対策. **看護学雑誌**, 68(1):10–18, 2004.

[11] 川越雅弘, 備酒伸彦, and 森山美知子. 要介護高齢者に対する退院支援プロセスへのリハビリテーション職種の関与状況: 急性期病床，回復期リハビリテーション病床，療養病床間の比較. **理学療法科学**, 26(3):387–392, 2011.

[12] 島田裕之, 内山靖, and 加倉井周一. 21 か月間の縦断研究による虚弱高齢者の転倒頻度と身体機能変化との関係. **総合リハビリテーション**, 30(10):935–941, 2002.

[13] 三浦研, 川越雅弘, and 孔相権. 要介護度および施設種別からみた歩行・移動に関する実態とその環境整備に関する基礎的研究–同一地域におけるアンケート調査から. **生活科学研究誌**, 6, 2007.

[14] Mary E Tinetti and Chandrika Kumar. The patient who falls:" "it's always a trade-off". *JAMA*, 303(3):258–266, 2010.

[15] Anne Forster, Ruth Lambley, Jo Hardy, John Young, Jane Smith, John Green, and Eileen Burns. Rehabilitation for older people in long-term care. *The Cochrane Library*, 2009.

[16] 高戸仁郎, 植木章三, 島貫秀樹, and 芳賀博. 携帯型歩数計を用いた高齢者の歩行能力評価法の開発. **保健福祉学研究**, 2:22–30, 2004.

[17] MHLW. *Physical Activity Standards 2013 for Health Promotion (in Japanese)*. MHLW, 2013.

[18] 大須賀洋祐, 藪下典子, 清野諭, 大久保善郎, 鄭松伊, 根本みゆき, フィゲロアラファエル, and 田中喜代次. 高齢者の身体活動基準に相当する 1 日あたりの歩数. **体力科学**, 64(2):243–250, 2015.

[19] S Nicole Culos-Reed, Lynette Stephenson, Patricia K Doyle-Baker, and James A Dickinson. Mall walking as a physical activity option: results of a pilot project. *Canadian Journal on Aging*, 27(01):81–87, 2008.

[20] Diane Podsiadlo and Sandra Richardson. The timed "up & go": a test of basic functional mobility for frail elderly persons. *Journal of the American Geriatrics Society*, 39(2):142–148, 1991.

[21] Anne Shumway-Cook, Sandy Brauer, and Marjorie Woollacott. Predicting the probability for falls in community-dwelling older adults using the timed up & go test. *Physical Therapy*, 80(9):896–903, 2000.

[22] Stephanie C Petterson, Ryan L Mizner, Jennifer E Stevens, LEO Raisis, Alex Bodenstab, William Newcomb, and Lynn Snyder-Mackler. Improved function from progressive strengthening interventions after total knee arthroplasty: a randomized clinical trial with an imbedded prospective cohort. *Arthritis Care & Research*, 61(2):174–183, 2009.

[23] 島田裕之, 古名丈人, 大渕修一, 杉浦美穂, 吉田英世, 金憲経, 吉田祐子, 西澤哲, and 鈴木隆雄. 高齢者を対象とした地域保健活動における timed up & go test の有用性. **理学療法学**, 33(3):105–111, 2006.

[24] 橋立博幸 and 内山靖. 虚弱高齢者における timed "up and go" test の臨床的意義. **理学療法学**, 32(2):59–65, 2005.

[25] Yosuke Yamada, Keiichi Yokoyama, Risa Noriyasu, Tomoaki Osaki, Tetsuji Adachi, Aya Itoi, Yoshihiko Naito, Taketoshi Morimoto, Misaka Kimura, and Shingo Oda. Light-intensity activities are important for estimating physical activity energy expenditure using uniaxial and triaxial accelerometers. *European Journal of Applied Physiology*, 105(1):141–152, 2009.

[26] Jonghoon Park, Kazuko Ishikawa-Takata, Shigeho Tanaka, Yuko Mekata, and Izumi Tabata. Effects of walking speed and step frequency on estimation of physical activity using accelerometers. *Journal of Physiological Anthropology*, 30(3):119–127, 2011.

[27] 樋口博之, 綾部誠也, 進藤宗洋　and 吉武裕, and 田中宏暁. 加速度センサーを内蔵した歩数計による若年者と高齢者の日常活動量の比較. **体力科学**, 52:111–118, 2003.

[28] Jennifer A Schrack, Rachel Cooper, Annemarie Koster, Eric J Shiroma, Joanne M Murabito, W Jack Rejeski, Luigi Ferrucci, and Tamara B Harris. Assessing daily physical activity in older adults: Unraveling the complexity of monitors, measures, and methods. *The Journals of Gerontology Series A*, page glw026, 2016.

[29] Mark G. Bowden, Chitralakshmi K. Balasubramanian, Andrea L. Behrman, and Steven A. Kautz. Validation of a speed-based classification system using quantitative measures of walking performance poststroke. *Neurorehabilitation and Neural Repair*, 2008.

[30] Liang-Kung Chen, Li-Kuo Liu, Jean Woo, Prasert Assantachai, Tung-Wai Auyeung, Kamaruzzaman Shahrul

Bahyah, Ming-Yueh Chou, Liang-Yu Chen, Pi-Shan Hsu, Orapitchaya Krairit, et al. Sarcopenia in Asia: consensus report of the Asian Working Group for Sarcopenia. *Journal of the American Medical Directors Association*, 15(2):95–101, 2014.

[31] Alfonso J. Cruz-Jentoft, Jean Pierre Baeyens, Jürgen M. Bauer, Yves Boirie, Tommy Cederholm, Francesco Landi, Finbarr C. Martin, Jean-Pierre Michel, Yves Rolland, Stéphane M. Schneider, Eva Topinková, Maurits Vandewoude, and Mauro Zamboni. Sarcopenia: European consensus on definition and diagnosis: Report of the european working group on sarcopenia in older people. *Age and Ageing*, page afq034, 2010.

[32] S. Shinkai, S. Watanabe, S. Kumagai, Y. Fujiwara, H. Amano, H. Yoshida, and H. Shibata. Walking speed as a good predictor for the onset of functional dependence in a Japanese rural community population. *Age and Ageing*, 29(5):441–446, 2000.

[33] Y. Fujita, Y. Nakamura, J. Hiraoka, K. Kobayashi, K. Sakata, M. Nagai, and H. Yanagawa. Physical-strength tests and mortality among visitors to health-promotion centers in Japan. *Journal of Clinical Epidemiology*, 48(11):1349–1359, 1995.

[34] R A Keith, C V Granger, B B Hamilton, and F S Sherwin. The functional independence measure. *Advances in Clinical Rehabilitation*, 1:6–18, 1987.

[35] Marshal F. Folstein, Susan E. Folstein, and Paul R. McHugh. "Mini-mental state": a practical method for grading the cognitive state of patients for the clinician. *Journal of Psychiatric Research*, 12(3):189–198, 1975.

[36] S. Fukuhara and Y. Suzukamo. *Manual of the SF-8 Japanese version (in Japanese)*. Institute for Health Outcomes & Process Evaluation Research, Kyoto, 2004.

[37] Aki Tsuchiya, Shunya Ikeda, Naoki Ikegami, Shuzo Nishimura, Ikuro Sakai, Takashi Fukuda, Chisato Hamashima, Akinori Hisashige, and Makoto Tamura. Estimating an EQ-5D population value set: the case of Japan. *Health Economics*, 11(4):341–353, 2002.

[38] 池田俊也, 白岩健, 五十嵐中, 能登真一, 福田敬, 齋藤信也, and 下妻晃二郎. 日本語版 EQ-5D-5L におけるスコアリング法の開発. **保健医療科学**, 64(1):47–55, 2015.

32 Appendix

被験者登録番号	
割付群	□ グループ A　□ グループ B
施設名	
報告書作成日	（西暦）　年　月　日
報告書作成者名	印
責任医師名	印

作成日　第　版　月　月　日

〔記入上の注意〕

1. 記入は、黒色のボールペンで行う。
2. 訂正は、訂正前の記入内容が分かるように二重線を引き、訂正日（年月日）と訂正理由の記入と試験責任医師又は試験分担医師の捺印を行う。
3. 空欄には「記入漏れ」と区別するために、その理由（実施せず・未実施）または斜線を入れる。

【主要評価項目】

歩数 (一日の歩数を記録)　年　月

日	歩数/活動量	日	歩数/活動量	日	歩数/活動量
1	/	11	/	21	/
2	/	12	/	22	/
3	/	13	/	23	/
4	/	14	/	24	/
5	/	15	/	25	/
6	/	16	/	26	/
7	/	17	/	27	/
8	/	18	/	28	/
9	/	19	/	29	/
10	/	20	/	30	/
	/		/	31	/

TUG	秒	月　日

【副次的評価項目】

握力	kg	MMSE			
10m 最大歩行	秒	BI		EuroQOL	
	歩	FIM		SF-8	

【臨床所見】

脈拍	bpm
自覚症状	□なし　□あり (下記に詳細を記入)

被験者名： 様	検査者名：	日付

Barthel Index (研究者記録用)

食事	自立、自助具などの装着可、標準時間内に食べ終える	10 点
	部分介助 (たとえば、おかずを切って細かくしてもらう)	5 点
	全介助	0 点
車椅子からベッドへの移乗	自立、ブレーキ、フットレストの操作も含める	15 点
	軽度の部分介助または監視を要する	10 点
	座ることは可能であるがほぼ全介助	5 点
	全介助または不可能	0 点
整容動作	洗面、整髪、歯磨き、髭剃りなどが自立	5 点
	部分介助または不可能	0 点
トイレ動作	自立 (衣服の操作、後始末を含む)	10 点
	部分介助、体を支える、衣服、後始末に介助を要する	5 点
	全介助または不可能	0 点
入浴	自立	5 点
	全介助または不可能	0 点
歩行・車椅子の移動	45m 以上の歩行が可能 (自助具の使用可)	15 点
	45m 以上の介助歩行ができる (自助具可)	10 点
	歩行不能の場合、車椅子にて 45m 以上の操作可能	5 点
	上記以外	0 点
階段昇降	自立、手すり等の使用の有無は問わない	10 点
	介助あるいは監視を要する	5 点
	不能	0 点
更衣動作	自立、靴・ジッパー・装具の着脱を含む	10 点
	上記以外	0 点
排便コントロール	失禁なし、浣腸・座薬の取り扱いも可能	10 点
	時に失禁あり、浣腸・座薬の取り扱いに介助を要する者も含む	5 点
	上記以外	0 点
排尿コントロール	失禁なし、収尿器の取り扱いも可能	10 点
	時に失禁あり、収尿器の取り扱いに介助を要する者も含む。	5 点
	上記以外。	0 点
合計		点

被験者名： 様	検査者名：	日付

FIM（研究者記録用）

運動項目		
食事	口に運ぶ動作、咀嚼、嚥下を含めた食事動作	
整容	口腔ケア、整髪、手洗い、洗顔、髭剃り・化粧など	
清拭	風呂、シャワーなどで首から下（背中以外）を洗う	
更衣・上半身	腰より上の更衣および義肢装具の装着	
更衣・下半身	腰より下の更衣および義肢装具の装着	
トイレ動作	衣服の着脱、排泄後の清潔、生理用具の使用	
排尿管理	排尿の管理、器具や薬剤の使用を含む、失敗の頻度	
排便管理	排便の管理、器具や薬剤の使用を含む、失敗の頻度	
ベッド・椅子・車椅子	それぞれの間の移乗、起立動作を含む	
トイレ	便器へ（から）の移乗	
浴槽・シャワー	浴槽、シャワー室へ（から）の移乗	
歩行 (車椅子)	屋内での歩行 (車椅子移動)	
階段	12 − 14 段の階段昇降	
認知項目		
理解	聴覚または視覚によるコミュニケーションの理解	
表出	言語的または非言語的表現	
社会的交流	他患、スタッフなどとの交流、社会的状況への順応	
問題解決	日常生活上での問題解決、適切な決断能力	
記憶	日常生活に必要な情報の記憶	
合計		点

運動項目
7: 完全自立 (時間、安全性含め)
6: 修正自立 (時間がかかる、補助具使用、安全性配慮)
5: 監視・準備
4: 最小介助 (患者自身で 75% 以上)
3: 中等度介助 (患者自身で 50% 以上)
2: 最大介助 (患者自身で 25% 以上)
1: 全介助 (患者自身で 25% 未満)

認知項目
7: 完全自立 (時間、安全性含め)
6: 修正自立 (時間がかかる、補助具使用、安全性配慮)
5: 監視・準備 (患者自身で 90% 以上)
4: 最小介助 (患者自身で 75% 以上)
3: 中等度介助 (患者自身で 50% 以上)
2: 最大介助 (患者自身で 25% 以上)
1: 全介助 (患者自身で 25% 未満)

Mini-Mental State Examination（MMSE)

得点：30 点満点

検査日：２００　　年　　月　　日　　曜日　　施設名：＿＿＿＿＿＿＿＿

被験者：＿＿＿＿＿＿＿＿　男・女　　生年月日：明・大・昭　　年　　月　　日　　歳

プロフィールは事前または事後に記入します。　　検査者：＿＿＿＿＿＿＿＿

	質問と注意点	回　答	得　点
**1**（５点）<br>時間の<br>見当識	**「今日は何日ですか」**<br>＊最初の質問で、被験者の回答に複数の項目が含まれていてもよい。その場合、該当する項目の質問は省く。	日	０　１
	**「今年は何年ですか」**	年	０　１
	**「今の季節は何ですか」**		０　１
	**「今日は何曜日ですか」**	曜日	０　１
	**「今月は何月ですか」**	月	０　１
**2**（５点）<br>場所の<br>見当識	**「ここは都道府県でいうと何ですか」**		０　１
	**「ここは何市（＊町・村・区など）ですか」**		０　１
	**「ここはどこですか」**<br>（＊回答が地名の場合、この施設の名前は何ですか、と質問をかえる。正答は建物名のみ）		０　１
	**「ここは何階ですか」**	階	０　１
	**「ここは何地方ですか」**		０　１
**3**（３点）<br>即時想起	**「今から私がいう言葉を覚えてくり返し言ってください。『さくら、ねこ、電車』はい、どうぞ」**<br>＊テスターは３つの言葉を１秒に１つずつ言う。その後、被験者にくり返させ、この時点でいくつ言えたかで得点を与える。<br>＊正答１つにつき１点。合計３点満点。<br>**「今の言葉は、後で聞くので覚えておいてください」**<br>＊この３つの言葉は、質問５で再び復唱させるので３つ全部答えられなかった被験者については、全部答えられるようになるまでくり返す（ただし６回まで）。		０　１<br>２　３
**4**（５点）<br>計算	**「１００から順番に７をくり返しひいてください」**<br>＊５回くり返し７を引かせ、正答１つにつき１点。合計５点満点。<br>正答例：９３　８６　７９　７２　６５<br>＊答えが止まってしまった場合は「それから」と促す。		０１２<br>３４５
**5**（３点）<br>遅延再生	**「さっき私が言った３つの言葉は何でしたか」**<br>＊質問３で提示した言葉を再度復唱させる。		０１２３
**6**（２点）<br>物品呼称	時計（又は鍵）を見せながら**「これは何ですか？」**<br>鉛筆を見せながら**「これは何ですか？」**<br>＊正答１つにつき１点。合計２点満点。		０１２
**7**（１点）<br>文の復唱	**「今から私がいう文を覚えてくり返し言ってください。『みんなで力を合わせて綱を引きます』」**<br>＊口頭でゆっくり、はっきりと言い、くり返させる。１回で正確に答えられた場合１点を与える。		０　１
**8**（３点）<br>口頭指示	＊紙を机に置いた状態で教示を始める。<br>**「今から私がいう通りにしてください。右手にこの紙を持ってください。それを半分に折りたたんでください。そして私にください」**<br>＊各段階毎に正しく作業した場合に１点ずつ与える。合計３点満点。		０１２３
**9**（１点）<br>書字指示	**「この文を読んで、この通りにしてください」**<br>＊被験者は音読でも黙読でもかまわない。実際に目を閉じれば１点を与える。	裏面に質問有	０　１
**10**（１点）<br>自発書字	**「この部分に何か文章を書いてください。どんな文章でもかまいません」**<br>＊テスターが例文を与えてはならない。意味のある文章ならば正答とする。（＊名詞のみは誤答、状態などを示す四字熟語は正答）	裏面に質問有	０　１
**11**（１点）<br>図形模写	**「この図形を正確にそのまま書き写してください」**<br>＊模写は角が１０個あり、２つの五角形が交差していることが正答の条件。手指のふるえなどはかまわない。	裏面に質問有	０　１

９．「この文を読んで、この通りにしてください」

め　　と

「目を閉じてください」

１０．「この部分に何か文章を書いてください。どんな文章でもかまいません」

１１．「この図形を正確にそのまま書き写してください」

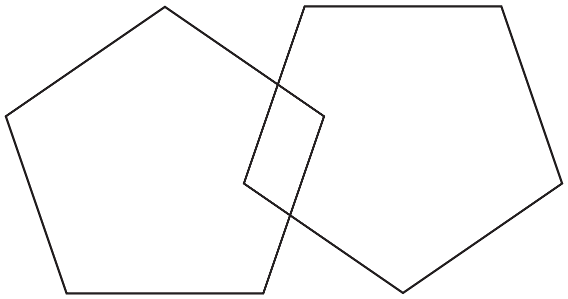

あなたの健康について

このアンケートはあなたがご自分の健康をどのように考えているかをおうかがいするものです。あなたが毎日をどのように感じ、日常の活動をどのくらい自由にできるかを知るうえで参考になります。お手数をおかけしますが、何卒ご協力のほど宜しくお願い申し上げます。

以下のそれぞれの質問について、一番よくあてはまるものに印（☑）をつけてください。

1.　全体的にみて、<u>過去１ヵ月間</u>のあなたの健康状態はいかがでしたか。

最高に良い	とても良い	良い	あまり良くない	良くない	ぜんぜん良くない
▼	▼	▼	▼	▼	▼
☐ 1	☐ 2	☐ 3	☐ 4	☐ 5	☐ 6

2.　<u>過去１ヵ月間</u>に、体を使う日常活動（歩いたり階段を昇ったりなど）をすることが身体的な理由でどのくらい妨(さまた)げられましたか。

ぜんぜん、妨(さまた)げられなかった	わずかに妨(さまた)げられた	少し妨(さまた)げられた	かなり、妨(さまた)げられた	体を使う日常活動ができなかった
▼	▼	▼	▼	▼
☐ 1	☐ 2	☐ 3	☐ 4	☐ 5

3.　<u>過去１ヵ月間</u>に、いつもの仕事（家事も含みます）をすることが、身体的な理由でどのくらい妨(さまた)げられましたか。

ぜんぜん、妨(さまた)げられなかった	わずかに妨(さまた)げられた	少し妨(さまた)げられた	かなり、妨(さまた)げられた	いつもの仕事ができなかった
▼	▼	▼	▼	▼
☐ 1	☐ 2	☐ 3	☐ 4	☐ 5

サンプル

SF-8™ Health Survey (Japanese version) Copyright © 1999, 2001, 2003 by QualityMetric, Inc. and Shunichi Fukuhara　All rights reserved.

4.　過去１ヵ月間に、体の痛みはどのくらいありましたか。

ぜんぜんなかった	かすかな痛み	軽い痛み	中くらいの痛み	強い痛み	非常に激しい痛み
☐ 1	☐ 2	☐ 3	☐ 4	☐ 5	☐ 6

5.　過去１ヵ月間、どのくらい元気でしたか。

非常に元気だった	かなり元気だった	少し元気だった	わずかに元気だった	ぜんぜん元気でなかった
☐ 1	☐ 2	☐ 3	☐ 4	☐ 5

6.　過去１ヵ月間に、家族や友人とのふだんのつきあいが、身体的あるいは心理的な理由で、どのくらい妨(さまた)げられましたか。

ぜんぜん、妨(さまた)げられなかった	わずかに、妨(さまた)げられた	少し、妨(さまた)げられた	かなり、妨(さまた)げられた	つきあいができなかった
☐ 1	☐ 2	☐ 3	☐ 4	☐ 5

7.　過去１ヵ月間に、心理的な問題（不安を感じたり、気分が落ち込んだり、イライラしたり）に、どのくらい悩まされましたか。

ぜんぜん悩まされなかった	わずかに悩まされた	少し悩まされた	かなり悩まされた	非常に悩まされた
☐ 1	☐ 2	☐ 3	☐ 4	☐ 5

8.　過去１ヵ月間に、日常行う活動（仕事、学校、家事などのふだんの行動）が、心理的な理由で、どのくらい妨(さまた)げられましたか。

ぜんぜん、妨(さまた)げられなかった	わずかに、妨(さまた)げられた	少し、妨(さまた)げられた	かなり、妨(さまた)げられた	日常行う活動ができなかった
☐ 1	☐ 2	☐ 3	☐ 4	☐ 5

ご協力、ありがとうございました。

サンプル

SF-8™ Health Survey (Japanese version) Copyright © 1999, 2001, 2003 by QualityMetric, Inc. and Shunichi Fukuhara　All rights reserved.

被験者名： 様	検査者名：	日付

EuroQOL (EQ-5D-3L) (研究者記録用)

移動の程度	私は歩き回るのに問題はない	1
	私は歩き回るのに問題がある	2
	私はベッド（床）に寝たきりである	3
身の回りの管理	私は身の回りの管理に問題はない	1
	私は洗面や着替えを自分でするのにいくらか問題がある	2
	私は洗面や着替えを自分でできない	3
ふだんの活動	私はふだんの活動を行うのに問題はない	1
（例：仕事、勉強、家族・余暇活動）	私はふだんの活動を行うのにいくらか問題がある	2
	私はふだんの活動を行うことができない	3
痛み/不快	私は痛みや不快感はない	1
	私は中程度の痛みや不快感がある	2
	私はひどい痛みや不快感がある	3
不安/ふさぎ込み	私は不安でもふさぎ込んでもいない	1
	私は中程度に不安あるいはふさぎ込んでいる	2
	私はひどく不安あるいはふさぎ込んでいる	3
結果<br>(結果は 11223 のように記す。)		

11111 1.000
11112 0.786
11113 0.736
11121 0.768
11122 0.705
11123 0.656
11131 0.654
11132 0.592
11133 0.542
11211 0.804
11212 0.742
11213 0.692
11221 0.724
11222 0.661
11223 0.612
11231 0.610
11232 0.548
11233 0.498
11311 0.715
11312 0.652
11313 0.603
11321 0.635
11322 0.572
11323 0.522
11331 0.521
11332 0.458
11333 0.409
12111 0.795
12112 0.732
12113 0.682
12121 0.714
12122 0.652
12123 0.602
12131 0.601
12132 0.538
12133 0.488
12211 0.751
12212 0.688
12213 0.638
12221 0.670
12222 0.608
12223 0.558
12231 0.557
12232 0.494
12233 0.444
12311 0.661
12312 0.599
12313 0.549
12321 0.581
12322 0.518
12323 0.469
12331 0.467
12332 0.405
12333 0.355
13111 0.747
13112 0.684
13113 0.634
13121 0.666
13122 0.604
13123 0.554
13131 0.553
13132 0.490
13133 0.440
13211 0.703
13212 0.640
13213 0.590
13221 0.622
13222 0.560
13223 0.510
13231 0.509
13232 0.446
13233 0.396
13311 0.614
13312 0.551
13313 0.501
13321 0.533
13322 0.470
13323 0.421
13331 0.419
13332 0.357
13333 0.307
21111 0.774
21112 0.711
21113 0.661
21121 0.693
21122 0.631
21123 0.581
21131 0.580
21132 0.517
21133 0.467
21211 0.730
21212 0.667
21213 0.617
21221 0.649
21222 0.587
21223 0.537
21231 0.536
21232 0.473
21233 0.423
21311 0.640
21312 0.578
21313 0.528
21321 0.560
21322 0.497
21323 0.448
21331 0.446
21332 0.384
21333 0.334
22111 0.720
22112 0.657
22113 0.608
22121 0.640
22122 0.577
22123 0.527
22131 0.526
22132 0.463
22133 0.414
22211 0.676
22212 0.613
22213 0.564
22221 0.596
22222 0.533
22223 0.483
22231 0.482
22232 0.419
22233 0.370
22311 0.587
22312 0.524
22313 0.474
22321 0.506
22322 0.444
22323 0.394
22331 0.393
22332 0.330
22333 0.280
23111 0.672
23112 0.609
23113 0.560
23121 0.592
23122 0.529
23123 0.479
23131 0.478
23132 0.415
23133 0.366
23211 0.628
23212 0.565
23213 0.516
23221 0.548
23222 0.485
23223 0.435
23231 0.434
23232 0.371
23233 0.322
23311 0.539
23312 0.476
23313 0.426
23321 0.459
23322 0.396
23323 0.346
23331 0.345
23332 0.282
23333 0.232
31111 0.430
31112 0.367
31113 0.318
31121 0.350
31122 0.287
31123 0.237
31131 0.236
31132 0.173
31133 0.124
31211 0.386
31212 0.323
31213 0.274
31221 0.306
31222 0.243
31223 0.193
31231 0.192
31232 0.129
31233 0.080
31311 0.297
31312 0.234
31313 0.184
31321 0.216
31322 0.154
31323 0.104
31331 0.103
31332 0.040
31333 -0.010
32111 0.376
32112 0.314
32113 0.264
32121 0.296
32122 0.233
32123 0.184
32131 0.182
32132 0.120
32133 0.070
32211 0.332
32212 0.270
32213 0.220
32221 0.252
32222 0.189
32223 0.140
32231 0.138
32232 0.076
32233 0.026
32311 0.243
32312 0.180
32313 0.131
32321 0.163
32322 0.100
32323 0.050
32331 0.049
32332 -0.014
32333 -0.063
33111 0.328
33112 0.266
33113 0.216
33121 0.248
33122 0.185
33123 0.136
33131 0.134
33132 0.072
33133 0.022
33211 0.284
33212 0.222
33213 0.172
33221 0.204
33222 0.141
33223 0.092
33231 0.090
33232 0.028
33233 -0.022
33311 0.195
33312 0.132
33313 0.083
33321 0.115
33322 0.052
33323 0.002
33331 0.001
33332 -0.062
33333 -0.111
